# Congruence as a measurement of extended haplotype structure across the genome

**DOI:** 10.1186/1479-5876-10-32

**Published:** 2012-02-27

**Authors:** Erin E Baschal, Jean M Jasinski, Theresa A Boyle, Pamela R Fain, George S Eisenbarth, Janet C Siebert

**Affiliations:** 1 Barbara Davis Center for Childhood Diabetes, University of Colorado Denver, Denver, CO 80045, USA; 2 CytoAnalytics, 1080 Bonnie Brae Blvd, Denver, CO 80209, USA

**Keywords:** Haplotype, Major histocompatibility complex, HapMap

## Abstract

**Background:**

Historically, extended haplotypes have been defined using only a few data points, such as alleles for several HLA genes in the MHC. High-density SNP data, and the increasing affordability of whole genome SNP typing, creates the opportunity to define higher resolution extended haplotypes. This drives the need for new tools that support quantification and visualization of extended haplotypes as defined by as many as 2000 SNPs. Confronted with high-density SNP data across the major histocompatibility complex (MHC) for 2,300 complete families, compiled by the Type 1 Diabetes Genetics Consortium (T1DGC), we developed software for studying extended haplotypes.

**Methods:**

The software, called ExHap (Extended Haplotype), uses a similarity measurement we term congruence to identify and quantify long-range allele identity. Using ExHap, we analyzed congruence in both the T1DGC data and family-phased data from the International HapMap Project.

**Results:**

Congruent chromosomes from the T1DGC data have between 96.5% and 99.9% allele identity over 1,818 SNPs spanning 2.64 megabases of the MHC (*HLA-DRB1 *to *HLA-A*). Thirty-three of 132 DQ-DR-B-A defined haplotype groups have > 50% congruent chromosomes in this region. For example, 92% of chromosomes within the DR3-B8-A1 haplotype are congruent from *HLA-DRB1 *to *HLA-A *(99.8% allele identity). We also applied ExHap to all 22 autosomes for both CEU and YRI cohorts from the International HapMap Project, identifying multiple candidate extended haplotypes.

**Conclusions:**

Long-range congruence is not unique to the MHC region. Patterns of allele identity on phased chromosomes provide a simple, straightforward approach to visually and quantitatively inspect complex long-range structural patterns in the genome. Such patterns aid the biologist in appreciating genetic similarities and differences across cohorts, and can lead to hypothesis generation for subsequent studies.

## Background

The structure of genetic variation across the human genome is complex and is characterized by blocks of extended linkage disequilibrium separated by recombination hotspots [1,2]. Extended haplotypes are one way to classify these regions of extended linkage disequilibrium. Historically, extended haplotypes have been defined using only a few data points, such as several HLA or complement genes in the major histocompatibility complex (MHC) [3-13], or 22 SNPs characterizing alleles of *CD28 *(7 SNPs), *ICOS *(8 SNPs), and *CTLA4 *(7 SNPs) on chromosome 2 [14]. High-density SNP data, and the increasing affordability of whole genome SNP typing, creates the opportunity to define higher resolution extended haplotypes. This drives the need for new tools that are able to support quantification and visualization of extended haplotypes as defined by as many as 2000 SNPs.

Several methods have been described to analyze the structure in dense SNP data including haplotype blocks and extended haplotype homozygosity (EHH). Haplotype block analyses, such as those implemented in the program Haploview [15], typically identify short-range linkage disequilibrium from < 1 kilobases (kb) to 173 kb [16]. EHH (the probability that two random chromosomes carrying a specific core haplotype are identical by state for all loci within a specified region) and related metrics have been used to identify regions of the genome that show evidence of recent positive selection as these regions are characterized by long range haplotypes [17-19].

However, as the number of nucleotides under consideration increases, a low level of allele mismatching must be accommodated to account for genotyping errors. Confronted with high-density SNP data across the MHC for 2,300 complete families, compiled by the Type 1 Diabetes Genetics Consortium (T1DGC), we defined a metric that we termed congruence which provides a way to quantify allele identity (identical by state alleles) across long genomic regions. We developed software, called ExHap (Extended Haplotype), which starts with phased chromosomes, derives a consensus string, identifies chromosomes that closely match this consensus, calculates overall congruence percentages, and computes allele identity of these congruent chromosomes.

In this field and in this text, the word chromosome is used in several different contexts. The first is in the general sense, e.g. "Human MHC Class 1 genes *HLA-A, HLA-B*, and *HLA-C *are found on chromosome 6." The second sense refers to a specific chromosome from an individual person as represented by some set of features, e.g. "In the HapMap data for chromosome 6, each specific chromosome is represented by 91,357 SNPs across the length of the chromosome" or "The T1DGC data set discussed in this work consists of 9280 chromosomes, represented by 2837 SNPs across the range 29.3 Mb to 34.2 Mb." Throughout this work, whenever we discuss specific chromosomes as represented by a set of features, we will clearly describe those features.

Using these concepts, we have shown that long, highly conserved haplotypes with identity of HLA alleles and hundreds to thousands of SNPs are frequent across the 4 megabases (Mb) of the MHC region [9-12]. In particular, the DR3-B8-A1 extended haplotype is a long-range, common haplotype that is associated with type 1 diabetes risk [10-13,20]. Chromosomes of this haplotype have a high level of allele identity across the MHC region with up to 9 Mb of near identity among chromosomes from apparently unrelated individuals [13].

Congruence quantifies allele identity across a large number of contiguous SNPs within a group of chromosomes. In this context, chromosome refers to a specific phased chromosome from an individual person, as represented by a set of SNPs. Here we apply ExHap to study congruence on 9,280 founder chromosomes genotyped for 2,837 SNPs across the MHC on chromosome 6p21. We also employ a sliding window to compute rolling short range congruence both within the MHC region and across the genome. Using these approaches, we compare overall congruence analyzing 1,818 SNPs in over 100 extended haplotype groups (defined by *HLA-DQB1, HLA-DRB1, HLA-B *and *HLA-A *alleles) from T1DGC data, illustrate statistically significant differences in congruence between haplotype groups, and inspect unrelated HapMap populations for congruence regions in a dataset of nearly 1.4 million SNPs across the human genome. Additionally, we compare the features and numeric results of ExHap to those of GERMLINE and Sweep, two other tools for identifying features of haplotype matrices.

## Methods

### Study populations and genotyping

This analysis included 2,300 affected sibling pair families (10,012 individuals typed for HLA and/or SNPs) from the Type 1 Diabetes Genetics Consortium (T1DGC), using the 2007.11.MHC data freeze [21]. Affected sibling pairs and their parents were enrolled in 9 cohorts worldwide. Within the analyzed cohorts of Asia-Pacific, Europe, North America, UK, British Diabetes Association (BDA), Danish, Human Biological Data Interchange (HBDI), Joslin and Sardinian, 99% of individuals are classified as white/Caucasian or unknown. The T1DGC performed basic quality control analyses on the data. All study participants or their parents/surrogates provided written informed consent to participate, and the study protocol was approved by the relevant Ethics Committees and Institutional Review Boards.

Genotyping was completed for 3,072 SNPs at the Wellcome Trust Sanger Institute, using two custom Illumina mapping panels [2957 distinct SNPs (1536 SNPs in each panel with 115 overlapping SNPs) with 2837 of 2957 SNPs successfully typed, yielding a 96% SNP success rate]. SNP positions ranged from 29.3 Mb to 34.2 Mb from the telomere, covering a range of approximately 4.9 Mb. SNPs were selected by the T1DGC using a tagging approach to account for the linkage disequilibrium in the region [21]. In addition, complete 4 digit HLA typing (*HLA-DPB1, HLA-DPA1, HLA-DQB1, HLA-DQA1, HLA-DRB1, HLA-B, HLA-C*, and *HLA-A*), performed using immobilized probe linear arrays, was available for all samples [22].

Data from the International HapMap Project (HapMap3, Release 2) was also used in this study. We studied 1,387,466 SNPs across the 22 autosomes. Phased founder chromosomes, as represented by SNPs across the length of the chromosomes, were used from both CEU (N = 176, CEPH, Utah residents with ancestry from northern and western Europe) and YRI (N = 200, Yoruba from Ibadan, Nigeria). For the Yoruba, donors were required to have four Yoruba grandparents. The criteria used to assign membership in the CEPH population have not been specified except that all donors were residents of Utah.

### Data processing

SNP positions used NCBI Build 36. T1DGC chromosomes (as represented by 2,837 SNPs in the approximately 4.9 Mb region located 29.3 Mb to 34.2 Mb from the telomere) were generated from SNP and HLA genotype data using multiple software packages. First, to establish that the genotype data demonstrated a Mendelian inheritance pattern within each family, the PedCheck program [23] was used on data from both Illumina panels and HLA separately. Mendelian inheritance patterns were present for all families. Next, data from the each of the two Illumina mapping SNP panels and the HLA panel were combined using custom Java programs. Merlin software [24] was used to phase the SNP and HLA genotype data from families into chromosomes. In situations of ambiguous phase (resulting from heterozygous SNPs or HLA in all family members), phase was not inferred. Instead, unphased alleles were labeled as such. Founder chromosomes were used in these analyses, yielding 4 unique chromosomes per family, for a total of 9,280 founder chromosomes. AFBAC (affected family based control) methodology was used to assign case or control status to founder chromosomes using Microsoft Excel macros as previously described [8,20,25,26].

### Haplotype groups

We analyzed founder MHC chromosomes from the T1DGC dataset, as represented by 1,818 SNPs ranging from *HLA-DRB1 *to *HLA-A *(2.64 Mb). We identified 132 groups of chromosomes having at least 10 chromosomes per group and identical *HLA-DQB1, HLADRB1, HLA-B*, and *HLA-A *alleles, hereafter called a DQ-DR-B-A "haplotype group." *HLADRB1 *and *HLA-DQB1 *are in strong linkage disequilibrium; therefore in this manuscript we refer to certain *DRB1*-*DQB1 *allele pairs by the *DRB1 *allele only. Hereafter, DRB1*01DQB1*05 will be referred to as DRB1*01, DRB1*03-DQB1*02 as DRB1*03, DRB1*04DQB1*0302 as DRB1*04, DRB1*07-DQB1*02 as DRB1*07, DRB1*08-DQB1*04 as DRB1*08, DRB1*11-DQB1*0301 as DRB1*11, DRB1*12-DQB1*0301 as DRB1*12, DRB1*15-DQB1*0602 as DRB1*15, and DRB1*16-DQB1*05 as DRB1*16 unless otherwise specified.

### Algorithm and software

**Figure 1:**
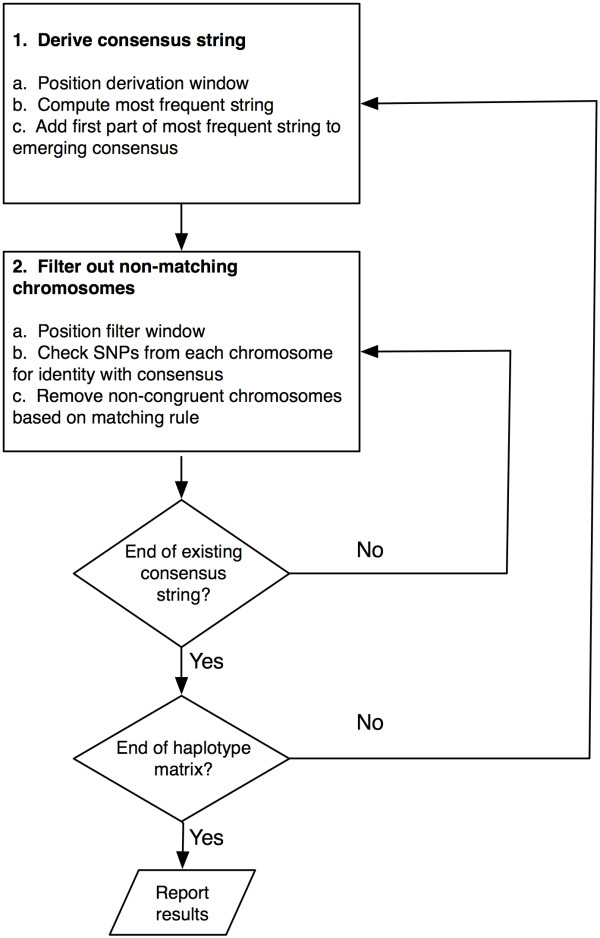
**Flow chart of ExHap algorithm**. This figure provides a high-level conceptual overview of the congruence finding algorithm. First, a consensus string is identified for a short section of the chromosomes under consideration. Second, chromosomes which do not match this consensus are eliminated from further consideration. The process repeats until the end of the overall region is reached

**Figure 2:**
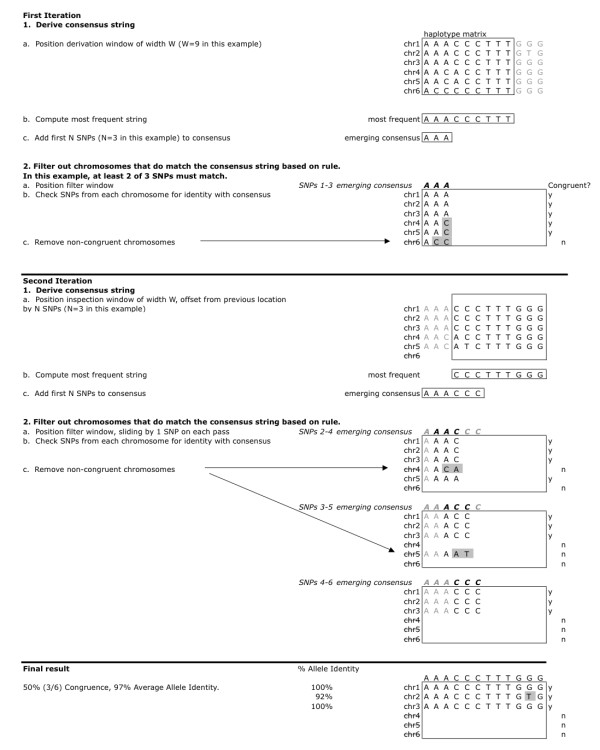
**An illustration of two iterations of ExHap**. This figure illustrates the first two iterations and the final result of ExHap on a simplified example. Each iteration consists of 2 parts: derivation of the consensus string and filtering of chromosomes for congruence. In Iteration 1, Part 1, we compute the most frequent sequence or string of 9 SNPs from our haplotype matrix. Then we add the first 3 SNPs from this string to the emerging consensus string. In Part 2, we filter the chromosomes based on whether or not each matches the consensus string using a matching rule. In this example, the matching rule is that at least 2 of 3 contiguous SNPs must match. SNPs that do not match are highlighted in gray. In Iteration 2, Part 1, the position of the derivation window is adjusted by 3 SNPs. In Part 2, we compare SNPs 2-6 of each chromosome to SNPs 2-6 of the consensus by inspecting 3 SNPs at a time. Thus, we must inspect SNPs 2-4, 3-5, and 4-6. The processes described here are repeated over the entire haplotype matrix. The final result shows the congruent chromosomes and the percentage of allele identity for each chromosome. The size of the derivation window and the number of SNPs to record are user-configurable. In this example, the size of the derivation window is 9 and the number of SNPs to record is 3. The size of the filter window, the number of SNPs that must match in this window, and the window offset are also user-configurable. In this example, the size of the filter window is 3, the number of matching SNPs is 2, and the offset is 1

Given a set of chromosomes represented by a range of contiguous SNPs, ExHap derives a single consensus string that captures the commonalities among chromosomes. It also identifies congruent chromosomes (those chromosomes that are identical to the consensus string for multiple overlapping blocks for at least 20 out of every 30 contiguous SNPs) and the percentage of allele identity between each chromosome and the consensus string. ExHap has two main parts: deriving a consensus string and filtering out chromosomes that do not match this string. The program (1) derives a consensus string by iteratively (a) positioning a derivation window of length W; (b) computing the most frequent string of length W within the derivation window; (c) appending the first N alleles of this most frequent substring to the consensus string; and (2) filters out chromosomes that do not match the emerging consensus sequence by (a) positioning the filter window; (b) within the filter window, checking SNPs from each chromosome for identity with the consensus sequence; and (c) removing noncongruent chromosomes from further consideration based on a matching rule for allele identity. A flow chart and simplified example is shown in Figures 1 and 2.

This algorithm detects allele identity across a large number of contiguous SNPs. The derivation window size, as specified by W, provides a "look ahead," favoring the selection of sequences that are identical beyond the recorded substring of length N. Commonly, we set W = 30 and N = 10. Our standard, but configurable, matching rule is that 20 of every 30 contiguous SNPs must match the consensus string. Unknown/unphased SNPs are not counted as mismatched. However, chromosomes with a large number of unknown/unphased SNPs can be excluded from analysis. A detailed description of the program and the userconfigurable parameters can be found in Additional file 1. ExHap is written in Java and is freely available as Additional file 2.

### GERMLINE and Sweep

Using GERMLINE version 1.4.2 [27], we generated pairwise shared segments over 3 different regions on 3 different chromosomes for both CEU and YRI cohorts from HapMap data as detailed above. We set minimum segment length (min_m) to 0.75 Mb. Additionally, we used the option to match segments on haplotypes rather than genotypes (h_extend). Using Sweep version 1.1 [17], we characterized the above-mentioned chromosomal regions with the ExportEHHvsFreqData batch command. We configured Sweep to compute EHH for each core haplotype at 600 Kb (MatchAt = Distance 600 Kb). Cores were configured using the default criteria of longest non-overlapping haplotype blocks as defined by Gabriel [16], with block length running from 3 to 20 SNPs.

### Recombination rates

To compare congruence to recombination rates, we used HapMap recombination rates, estimated from phased haplotypes in HapMap Release 22 (NCBI Build 36). These rates were computed from the pooled populations of the CEU, YRI, and JPT + CBT. For each 250 SNP region for which we computed congruence, we also identified the maximum recombination rate. For chromosome 10, we plotted the percent congruence versus this maximum recombination rate, identifying multiple regions in which both congruence rates and recombination rates were relatively high.

Identification of known genes and associated KEGG pathways in regions of candidate extended haplotypes.

For each candidate extended haplotype listed in Additional file 3: Table S2 (e.g. Cohort YRI, chromosome 1, starting position 45708938, ending position 46703019), we queried the UCSC Genome Browser database [28]http://genome.ucsc.edu through the MySQL interface, issuing queries using the following template: "select distinct 'YRI', chrom, txStart, geneSymbol, description from hg18.knownGene k, hg18.kgXref x where k.name = x.kgID and txStart > 45708938 and txEnd < 46703019 and chrom = 'chr1' order by txStart;" For each unique resulting gene, we then issued a query against the KEGG pathway tables using the following query template: "select distinct km.description from hg18.knownGene k, hg18.kgXref x, hg18.keggMapDesc km, hg18.keggPathway kp where k.name = x.kgID and x.kgID = kp.kgID and kp.mapID = km.mapID and geneSymbol = 'AARSD1';".

### Statistical analysis

The Wilcoxon signed-rank test was used to compare congruence percentages between groups across chromosomal ranges, with a significance level of α = 0.05. All statistical computations were performed using the R Project for Statistical Computing [29]. Heat map style allele identity plots were generated with the heatmap.2 package in R.

Linkage disequilibrium plots, haplotype blocks, and associated metrics were generated with Haploview version 4.2 [15]. Blocks were determined using the default Gabriel et al. criteria, ignoring pairwise comparisons of markers greater than 500 kb apart [16].

## Results

### Differing levels of congruence across haplotype groups in the T1DGC

We inspected phased HLA and SNP data on chromosome 6, spanning the range *HLA-DRB1 *to *HLA-A *(32.6 Mb to 30.0 Mb, 1818 SNPs across 2.64 Mb). Hereafter this data will be referred to as a chromosome. We stratified the T1DGC MHC founder chromosomes into haplotype groups defined by identity of DQ-DR-B-A as described in the methods. We restricted analysis to haplotype groups containing at least 10 chromosomes, resulting in 132 HLA "identical" groups identified for further study. Using ExHap, we identified congruent chromosomes for each of these haplotype groups. Details of the algorithm are provided in the methods and illustrated in Figures 1 and 2. Briefly, ExHap identifies the most common string of SNPs over a small window or subrange of, for example, 30 SNPs. Then, ExHap slides the window a smaller number of SNPs (e.g. 10) and repeats the process, thereby deriving a consensus string for the entire range. Additionally, as the consensus string emerges, ExHap scores each chromosome for identity with the emerging string. Chromosomes that do not sufficiently match the consensus are eliminated from further consideration.

**Figure 3:**
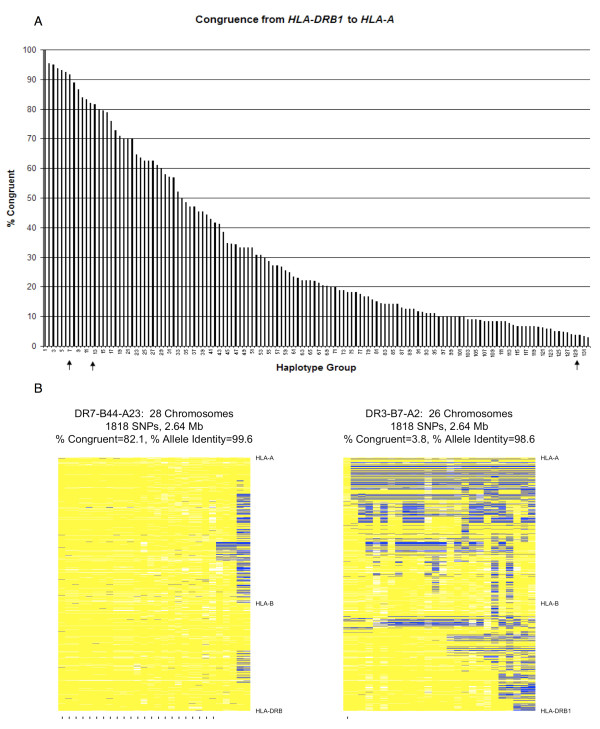
**Congruence is widespread across the MHC and is not limited to the DR3-B8A1 haplotype**. **A**: Percent congruence of 132 extended DQ-DR-B-A haplotype groups from *HLA-DRB1 *to *HLA-A*. Plotted is the percent of chromosomes (both cases and controls) within each HLA haplotype group that meet the rules for congruence from *HLA-DRB1 *to *HLA-A*. The number of chromosomes in each group ranged from 8 to 747 (mean = 34, median = 17), and specific details for each haplotype group are given in Additional file 4: Table S1. Arrows identify haplotype groups that will be discussed further in the paper (number = DR.B.A: 7 = 3.8.1, 12 = 7.44.23, 129 = 3.7.2). **B**: Examples of congruence within two haplotype groups. In these two images, each column represents one founder chromosome (represented by 1,818 SNPs across 2.64 Mb ranging from *HLA-DRB1 *to *HLA-A *on chromosome 6) with the specified HLA alleles (e.g., DR7, B44 and A23). Each row illustrates data for one SNP. Yellow represents alleles that match the consensus sequence, whereas blue represents alleles that do not match the consensus. White denotes missing or unphased alleles. Congruent chromosomes are indicated by a tick mark at the bottom of the image. The DR7-B44-A23 haplotype group exhibits extreme congruence from *HLA-DRB1 *to *HLA-A*, seen by the high frequency of chromosomes that are congruent for the entire region (yellow from *HLA-DRB1 *to *HLA-A*). In contrast, DR3-B7-A2 shows an example of a haplotype group in which only one of the chromosomes is congruent across the region. Because the consensus chromosome is derived from a set of chromosomes, it is not necessarily an exact match to any particular chromosome. Thus the one congruent chromosome in this haplotype group does not have 100% allele identity

Figure 3A illustrates congruence for each DQ-DR-B-A haplotype group. Our results identify 33 haplotype groups with over 50% congruence. This means that at least half of their chromosomes are nearly identical in the region between *HLA-DRB1 *and *HLA-A*. In particular, the common 3.8.1 (DR3-B8-A1) haplotype is highly congruent from *HLA-DRB1 *to *HLA-A *[91.7% (685/747 chromosomes)]. Across 132 MHC haplotype groups, average allele identity for congruent chromosomes within a haplotype group varies from 96.5% to 99.9%, with a median of 99.5%, over 2.64 megabases. Detailed congruence results for all haplotype groups are included in Additional file 4: Table S1. Two examples of chromosome level patterns of allele identity are shown in Figure 3B, the highly congruent DR7-B44-A23 (23/28 chromosomes, 82% congruent from *HLA-DRB1 *to *HLA-A*) and the less congruent DR3-B7-A2 (1/26 chromosomes, 4% congruent). In these two images (what we will call allele identity plots), each column represents one founder chromosome (as represented by 1,818 SNPs across the 2.64 Mb range) with the specified HLA alleles (e.g., DR7, B44 and A23) and each row represents one SNP. Yellow represents alleles that match the consensus sequence whereas blue represents alleles that do not match the consensus. Eighty-two percent of the DR7-B44-A23 chromosomes are congruent, while only one of the DR3-B7-A2 chromosomes is congruent. By definition, at least 1 chromosome must be congruent, so this represents the lower limit of congruence. Chromosomes congruent for the entire region are shown on the left as indicated by the tick mark below the chromosome. ExHap generates output preformatted for these "heat map" style graphics.

### Rolling congruence in the T1DGC

**Figure 4:**
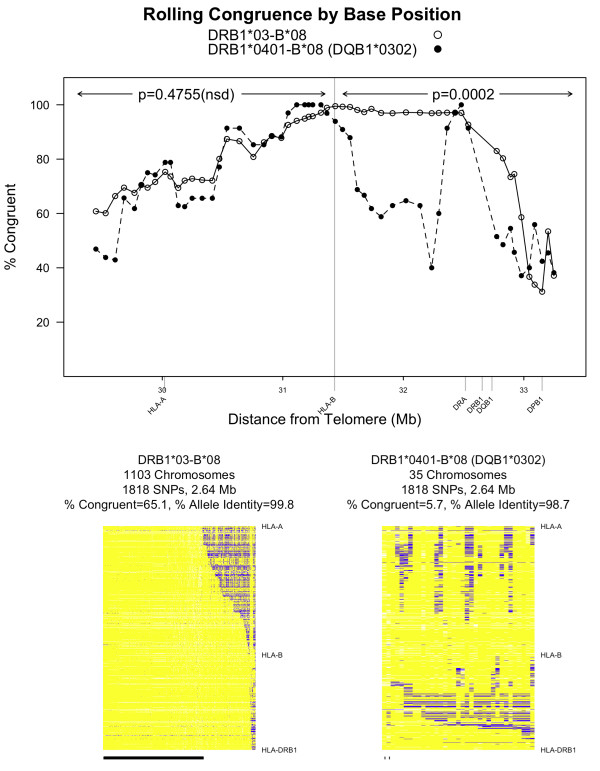
**Comparison of two haplotype groups from the T1DGC**. **A**: Congruence was calculated separately for two haplotype groups, DRB1*03-B*08 and DRB1*0401-B*08 (DQB1*0302). There is a significant difference in the congruence between the two groups centromeric of *HLA-B*, while the congruence within the two groups is not significantly different telomeric of *HLA-B*. **B**: In these two images, each column illustrates one founder chromosome (represented by 1,818 SNPs across 2.64 Mb ranging from *HLA-DRB1 *to *HLA-A *on chromosome 6) with the specified HLA alleles (e.g., DRB1*03 and B*08). Each row illustrates data for one SNP. Yellow represents alleles that match the consensus sequence, whereas blue represents alleles that do not match the consensus. White denotes missing or unphased alleles. Congruent chromosomes are indicated by a tick mark at the bottom of the image. The DRB1*03-B*08 chromosomes are highly congruent across the entire region from *HLA-DRB1 *to *HLA-B*, and in many cases from *HLA-DRB1 *to *HLA-A*. On the other hand, the DRB1*04-B*08 (DQB1*0302) chromosomes are congruent around *HLA-B *but are not congruent near *HLA-DRB1 *or *HLA-A*

In addition to examining congruence across a specific range of SNPs (*HLA-DRB1 *to *HLA-A)*, we inspected "rolling congruence." Figure 4A shows rolling congruence of two different haplotype groups, DRB1*03-B*08 and DRB1*0401-B*08 (DQB1*0302). We computed congruence for contiguous sliding windows of 250 SNPs, with each window offset from the previous window by 50 SNPs (e.g., SNPs 1-250, 50-300, 100-350 etc.), resulting in 55 observations across the MHC. The percentage of congruent regions (as defined by the 250 SNPs under consideration) is plotted for each window. As can be seen in Figure 4A, the regions on DRB1*03-B*08 chromosomes are significantly more congruent from *HLA-DRB1 *to *HLA-B *than the regions on DRB1*0401-B*08 (DQB1*0302) chromosomes even though each group is fixed for both *HLA-DRB1 *and *HLA-B *alleles (*p *= 0.0002, Wilcoxon signed-rank test, mean congruence = 81.5% for DRB1*03-B*08 compared to mean congruence = 63.8% for DRB1*0401-B*08). These differences can be visualized by examining the allele identity plots for these two groups in Figure 4B (same color coding as Figure 3B). For the most part, the DRB1*03-B*08 chromosomes are extremely congruent from *HLA-DRB1 *to *HLA-B *(96% of chromosomes are congruent), with 65% (719/1103) of the chromosomes congruent all the way from *HLA-DRB1 *to *HLA-A*. The DRB1*0401-B*08 (DQB1*0302) chromosomes, on the other hand, are congruent in the central region, near *HLA-B*, but are not congruent near *HLA-DRB1 *or *HLA-A*. We can see from these examples that fixing *HLADRB1 *and *HLA-B *alleles is not sufficient to guarantee congruence, and that the DRB1*03B*08 chromosomes are strikingly more congruent than the DRB1*0401-B*08 (DQB1*0302) chromosomes across the analyzed regions.

### Rolling congruence in HapMap

Motivated by results from the MHC region, we applied ExHap to family-phased data from two HapMap populations [CEU (CEPH, Utah residents with ancestry from northern and western Europe) and YRI (Yoruba from Ibadan, Nigeria)] across the 22 autosomes (Additional file 5: Figure S1). We computed congruence for contiguous sliding windows of 250 SNPs, with each window offset from the previous window by 50 SNPs, resulting in 27,652 observations across the genome. In this case, chromosomes are represented by 20,085 to 116,415 SNPs (chromosome 22 and 1, respectively). Congruence metrics are based on 250 contiguous SNPs. As expected, congruence in the CEU cohort is higher than in the YRI cohort, with the CEU congruence exceeding YRI congruence for 72% of the observations (19,914/27,652). Furthermore, for the CEU, the 99^th ^percentile of these observations was 51.1%, as compared to 27.5% for YRI. To identify candidate extended haplotypes, we looked for two or more contiguous observations that exceeded the 99^th ^percentile for the respective population. We identified 51 such candidate extended haplotypes in CEU and 55 in YRI. These candidate extended haplotypes are at least 300 SNPs in length, and are detailed in Additional file 3: Table S2.

To provide a bit more detail and context about these candidate extended haplotypes, we identified genes contained in the regions and associated pathways. For each cohort, for each candidate extended haplotype, we queried the UCSC Genome Browser database to first identify known genes in these regions. Then, for each cohort, for each gene, we queried the database to identify KEGG pathways with which the gene is associated. We identified 900 distinct known genes in the candidate extended haplotype regions of the CEU cohort, and 1043 in the YRI cohort. In the CEU data, the 900 genes resulted in a total of 428 hits in the KEGG pathways across 134 distinct pathways. In the YRI data, the 1043 genes resulted in 750 hits in the KEGG pathways across 165 distinct pathways. All resulting genes are listed in Additional file 6: Table S3. All resulting pathways, together with the number of associated genes, are listed in Additional file 7: Table S4. The pathways with which 10 or more genes from the YRI cohort are associated are summarized in Table 1.

**Table 1:** Extract of Pathway Table.

Pathways	CEU Hits	YRI Hits
Olfactory transduction	11	56

Systemic lupus erythematosus	15	49

Metabolic pathways	36	47

MAPK signaling pathway	12	20

Regulation of actin cytoskeleton	9	14

Cytokine-cytokine receptor interaction	2	13

Endocytosis	5	11

Cell cycle	9	10

Glycolysis/Gluconeogenesis	3	10

Antigen processing and presentation	2	10

Chemokine signaling pathway	1	10

The following pathways were associated with 10 or more genes found in candidate extended haplotypes of the YRI cohort. In all cases shown here, there are more associated genes in the YRI cohort than in the CEU cohort. This is an extract of Additional file 7: Table S4

**Figure 5:**
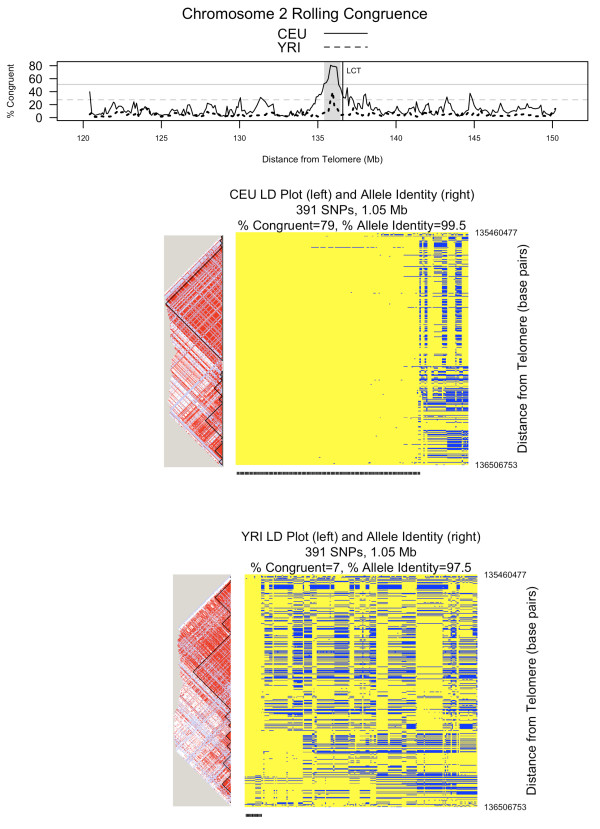
**Comparing unrelated HapMap populations: chromosome 2**. This figure shows rolling congruence over a section of chromosome 2. Detailed linkage disequilibrium (LD) plots with haplotype blocks and allele identity plots correspond to the shaded peak. The two horizontal reference lines on the rolling congruence plot correspond to the 99^th ^percentile of congruence observations across the genome. That value is 51.1% for CEU (solid line) and 27.5% for YRI (dotted line). On the LD plots, shades of red indicate increasing D'. For the allele identity plots, each column is one chromosome (as represented by 391 SNPs and 1.05 Mb) and each row is a SNP. Yellow represents alleles that match the consensus sequence whereas blue represents alleles that do not match the consensus. Congruent chromosomes are indicated by the tick mark at the bottom of the allele identity plot. This peak on chromosome 2 represents a region where CEU chromosomes are more congruent than YRI chromosomes and is near the *LCT *gene, previously implicated as a target of recent positive selection

**Figure 6:**
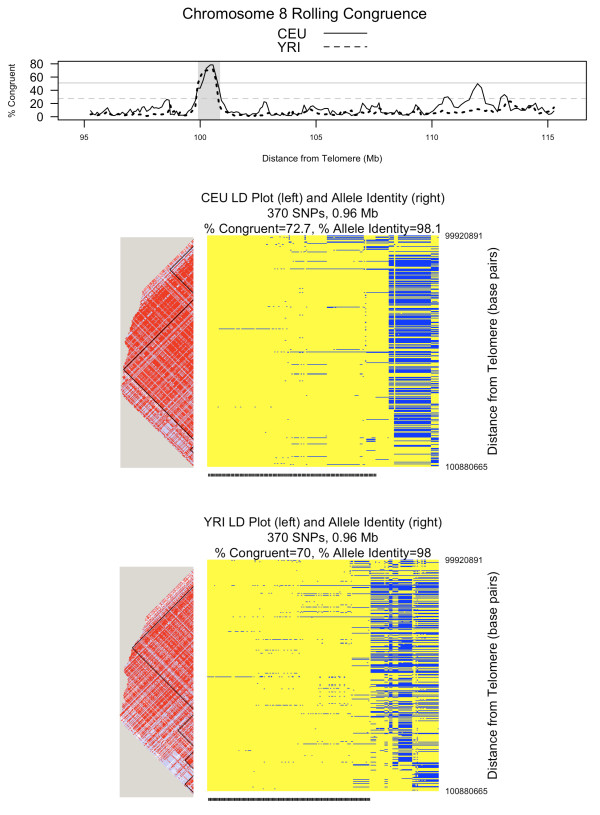
**Comparing unrelated HapMap populations: chromosome 8**. This figure shows rolling congruence over a section of chromosome 8. Detailed linkage disequilibrium (LD) plots with haplotype blocks and allele identity plots correspond to the shaded peak. The two horizontal reference lines on the rolling congruence plot correspond to the 99^th ^percentile of congruence observations across the genome. That value is 51.1% for CEU (solid line) and 27.5% for YRI (dotted line). On the LD plots, shades of red indicate increasing D'. For the allele identity plots, each column is one chromosome (as represented by 370 SNPs and 0.96 Mb) and each row is a SNP. Yellow represents alleles that match the consensus sequence whereas blue represents alleles that do not match the consensus. Congruent chromosomes are indicated by the tick mark at the bottom of the allele identity plot. This peak on chromosome 8 is characterized by both high CEU and high YRI congruence

**Figure 7:**
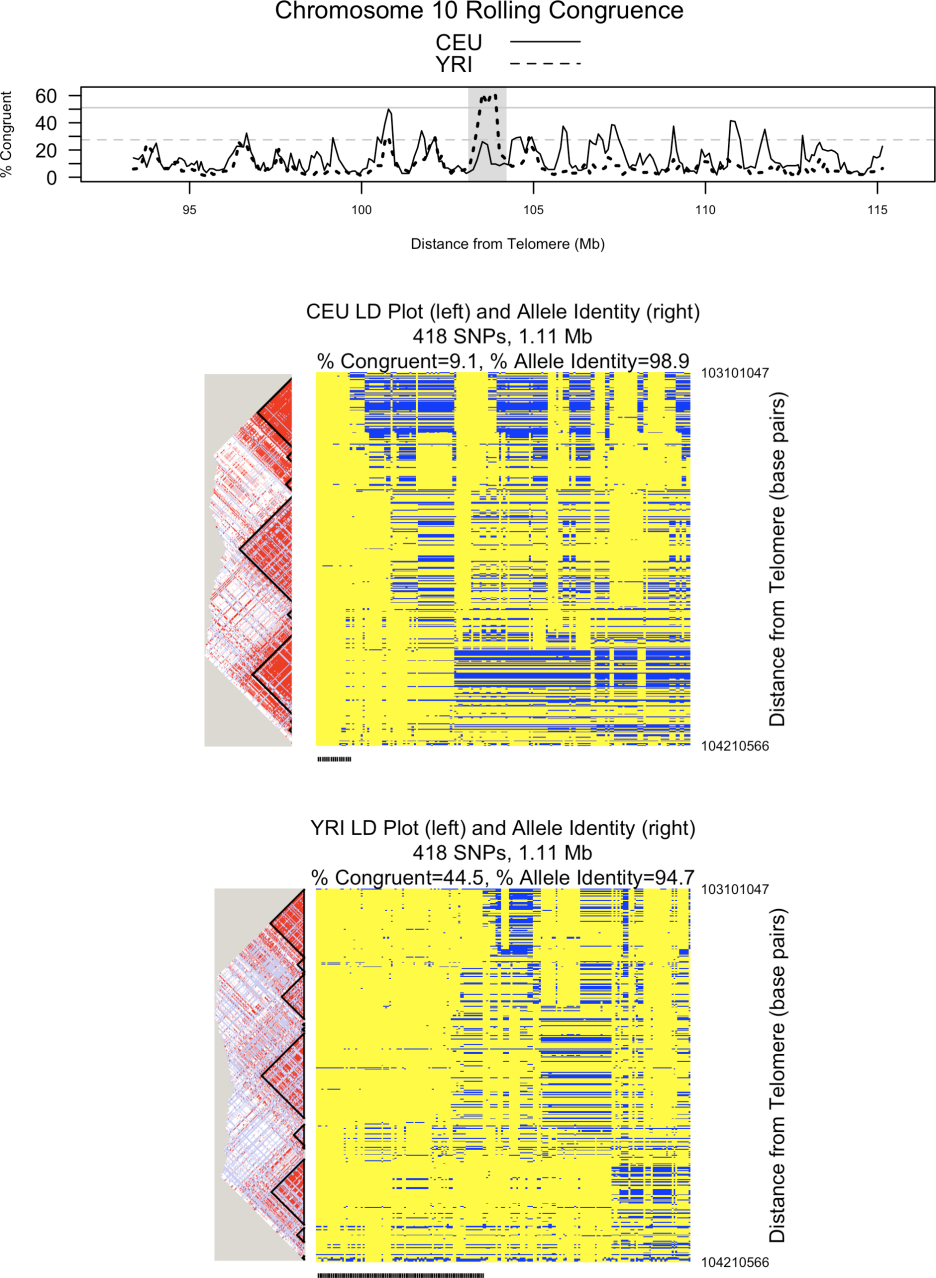
**Comparing unrelated HapMap populations: chromosome 10**. This figure shows rolling congruence over a section of chromosome 10. Detailed linkage disequilibrium (LD) plots with haplotype blocks and allele identity plots correspond to the shaded peak. The two horizontal reference lines on the rolling congruence plot correspond to the 99^th ^percentile of congruence observations across the genome. That value is 51.1% for CEU (solid line) and 27.5% for YRI (dotted line). On the LD plots, shades of red indicate increasing D'. For the allele identity plots, each column is one chromosome (as represented by 418 SNPs and 1.11 Mb) and each row is a SNP. Yellow represents alleles that match the consensus sequence whereas blue represents alleles that do not match the consensus. Congruent chromosomes are indicated by the tick mark at the bottom of the allele identity plot. This peak on chromosome 10 (near 103 Mb) demonstrates high YRI congruence but lower CEU congruence

Figures 5, 6, and 7 show rolling congruence, allele identity, and linkage disequilibrium plots for three representative candidate extended haplotypes. The region highlighted on chromosome 2 shows a high congruence peak for CEU only (Figure 5), the region on chromosome 8 for both CEU and YRI (Figure 6), and the region on chromosome 10 for YRI only (Figure 7). As can be seen from the allele identity plots for each of these regions, high levels of congruence correspond to high levels of allele identity as indicated by the yellow regions in the allele identity plots. The peak on chromosome 2 (Figure 5), where the CEU chromosomes are more congruent than the YRI chromosomes, includes the lactase gene (*LCT*). Strong recent positive selection has been reported for a large region that includes *LCT *[17-19]. The peak on chromosome 8 is characterized by both high CEU and high YRI congruence, with the CEU and YRI consensus sequences matching for 367 of 370 SNPs (99%, Figure 6). The peak on chromosome 10 demonstrates high YRI congruence but lower CEU congruence (Figure 7). Interestingly, for this chromosome 10 region, the YRI population shows 44.5% congruence with a median multi-allelic D' of 0.75 (range 0.45-1.0) across 12 blocks. In contrast, the CEU population shows lower congruence at 9.1% but a higher median multi-allelic D' of 0.87 (range 0.33-0.98) across 13 blocks. Thus, congruence and haplotype block-associated metrics capture different features of the region.

### Comparison of ExHap to GERMLINE and Sweep

We compared the features and results of ExHap to those generated by GERMLINE [27] and Sweep [17]. All three approaches identify patterns of similarity in sets of phased chromosomes as defined by a series of SNPs. GERMLINE was designed to identify hidden relatedness as defined by long nearly identical regions shared by two chromosomes. Sweep was designed to identify regions of recent positive selection. ExHap was designed to identify both a consensus sequence and to score individual chromosomes for a configurable level of matching with that consensus. Because the three approaches were intended to highlight different characteristics of haplotype matrices, comparisons among them should be interpreted with caution. That said, Table 2 summarizes selected features of the approaches while Table 3 presents numeric results.

**Table 2:** Feature comparison of ExHap, GERMLINE and Sweep.

Feature	ExHap	GERMLINE	Sweep/EHH
Requires phased chromosomes as input	yes	yes	yes

Handles missing data	yes	no	no

Allows small number of mismatches (fuzzy matching)	yes	yes^1^	no

Designed to analyze a large number of SNPs (100 s to 1000 s)	yes	yes	no

Supports genetic distance	no	yes	yes

Outputs aggregate/region-wide metrics	yes	no^2^	yes^3^

Outputs chromosome level detail	yes	yes^4^	no

Seven features of starting data sets, algorithm logic, and resulting data are listed below. Each program is characterized based on the presence (yes) or absence (no) of the feature

^1^Initial "seed" "haplotypes" are exact match. After that, a user-configurable number of mismatches is allowed

^2^Segment-level details (shared between 2 chromosomes) can be aggregated to describe region

^3^Metrics are reported at a "core" level, where a core is a particular haplotype within a haplotype block. These metrics can be aggregated across all cores within a region to describe the region

^4^Primary output is overlapping segments shared between 2 chromosomes, but report is at an individual level rather than chromosome level

**Table 3:** Numeric comparison of ExHap, GERMLINE, and Sweep.

			ExHap	GERMLINE	Sweep
	**Range in base pairs**	**Cohort**	**Percent Congruet**	**Avg # of Shared Segments per chromosome, 0.75 Mb min length**	**Avg EHH of 30 Core Haplotypes with Highest EHH at 600 Kb From Core**

Chr2	135460477-136506753	CEU	79.0	19.1	0.69

		YRI	7.0	2.8	0.59

Chr8	99920891-100880665	CEU	72.7	9.3	0.90

		YRI	70.0	7.9	0.84

Chr10	103101047-104210566	CEU	9.1	4.5	0.51

		YRI	44.5	3.0	0.70

Three regions were selected based on patterns of congruence in HapMap CEU and YRI cohorts. Allele identity plots of the regions are presented in Figures 5, 6, and 7. GERMLINE results are expressed as average number of shared segments per chromosome to account for the difference in the number of chromosomes between CEU (n = 176) and YRI (n = 200)

ExHap handles missing data (unknown or unphased), while Sweep and GERMLINE require complete phasing, which can be inferred using programs such as BEAGLE and PHASE [30-33]. Both ExHap and GERMLINE allow a small number of mismatches. In part because of this tolerance for noise both ExHap and GERMLINE produce meaningful numeric results over longer distances than does Sweep. Because Sweep requires exact matches by design, Extended Haplotype Homozygosity (EHH) deteriorates with diversity, which all other factors being equal, is likely to increase with distance. Both GERMLINE and Sweep are able to compute and report results in either genetic distance or physical distance, while the current implementation of ExHap reports physical distance only. ExHap reports both aggregate region-wide metrics and chromosome level detail. In contrast, GERMLINE reports segments that are shared by pairs of chromosomes, while Sweep reports metrics at a core level.

Table 3 summarizes results from all 3 approaches for the regions on chromosomes 2, 8 and 10 illustrated in Figures 5, 6 and 7. To compare GERMLINE to ExHap, we used GERMLINE to compute the number of shared segments with a length of at least 0.75 Mb in the specified regions. Then, to account for the different number of chromosomes in the CEU and YRI data sets, we divided the total number of shared segments by the number of chromosomes to derive the average number of shared segments per chromosome. For the region on chromosome 2, both Percent Congruent and Average Number of Shared Segments are notably larger for CEU than YRI. For the region on chromosome 8, the Average Number of Shared Segments for CEU and YRI are relatively close, as are the Percent Congruent measurements. For the region on chromosome 10, the difference in Percent Congruent between CEU and YRI is much more dramatic than the difference in Average Number of Shared Segments. However, upon inspection of Figure 7, this is a relatively noisy region, which is reflected by the generally low average number of shared segments reported by GERMLINE.

To compare Sweep to ExHap, we used Sweep to identify the core haplotype blocks (as defined by Gabriel [16]) between 3 and 20 SNPs in length, and to calculate EHH for all haplotypes within each core block at a distance of 600 Kb. Finally, we computed the average EHH of the 30 haplotype cores with the highest EHH. This provides some degree of normalization for the different number of chromosomes in the two cohorts. Hereafter, we will refer to this metric as AvgEHH-30. The parameters of 600 Kb in distance and top 30 haplotype cores were selected after experimentation with a variety of parameter settings. By definition, EHH and thus AvgEHH-30 can range from 0 to 1. The smallest difference in AvgEHH-30 between CEU and YRI is 0.06 for the region on chromosome 8. This is also the region with the smallest difference in Percent Congruent as derived by ExHap. The largest difference in AvgEHH-30 is 0.19 for chromosome 10. YRI has both the larger Percent Congruent value and the larger AvgEHH-30. On chromosome 2, CEU has both the larger Percent Congruent value and the larger AvgEHH-30, though the difference in AvgEHH-30 is not as dramatic as the difference in Percent Congruent.

In summary, all three programs were designed to identify different phenomenon in haplotype matrices. Thus, the numeric results are not directly comparable. However, the discussion of features and results illustrates the many ways in which chromosomal regions can be studied.

## Discussion

We studied congruence across the MHC region using a large dataset from the Type 1 Diabetes Genetics Consortium (T1DGC) with HLA allele and SNP typing (2,837 SNPs) for both affected sibling pairs and their parents. We defined chromosomal phase with familial analysis. We note that there are 33 haplotype groups in the T1DGC dataset that are > 50% congruent (Figure 3A). In addition, we investigated the genome-wide congruence of family-phased founder chromosomes for two cohorts from HapMap. We identified 51 candidate extended haplotypes in the CEU data and 55 in the YRI data. Thus, it is plausible that biologically interesting extended haplotypes are located across the genome.

The MHC region contains many genes with known immunological function. When stratified by HLA alleles (DQ-DR-B-A), there are 132 haplotype groups across the MHC region. Within these groups, congruence ranges from 3% to 100%, with a mean of 32.8%. As demonstrated by these results, and as illustrated in Figure 3, the haplotype group alone does not guarantee a high level of congruence in dense SNP data.

We applied rolling congruence to two haplotype groups from the T1DGC (Figure 4). The DRB1*0401-B*08 chromosomes are significantly less congruent than the DRB1*03-B*08 chromosomes across the region from *HLA-DRB1 *to *HLA-B*. This offers an additional line of evidence that simply fixing *HLA-DRB1 *and *HLA-B *alleles is not sufficient to drive congruence within any particular haplotype group.

In the two HapMap populations analyzed, CEU and YRI, CEU exhibits higher congruence than YRI for 72% of our observations. YRI is an older population compared to CEU, and thus we expect YRI to demonstrate greater genetic diversity and less linkage disequilibrium than CEU. Therefore, regions where YRI has higher congruence than CEU are of particular interest, as they may reflect important underlying biological effects, especially with respect to survival in the tropics. The observation that the candidate extended haplotypes in the YRI cohort contain more genes associated with known KEGG pathways may also be related to the importance of these regions in long-term population survival. In addition, the MHC region on chromosome 6p21 does not appear to be uniquely congruent, as other genomic regions demonstrate similar levels of congruence.

High levels of congruence in regions across the genome could be explained by several mechanisms. First, the regions identified could simply represent regions that exhibit decreased recombination and therefore decreased haplotype diversity. While some regions of high congruence are also associated with low recombination, such as in the centromere, there are regions of high congruence and high recombination (Additional file 8: Figure S2). Second, the regions identified could be a result of strong negative selection. Third, the identified regions could be under recent positive selection. Regions of the genome that have been under recent positive selection are typically characterized by long-range haplotypes surrounding the mutation that provides the selective advantage and attains high frequency rapidly. A known recent selective event at the *LCT *locus is associated with the increase in animal domestication and adult milk consumption [17-19,34]. Our CEU data also shows a congruence peak at this locus.

Both haplotype block analysis and congruence analysis capture the patterns in haplotype matrices. Haplotype blocks work well for relatively small regions. When applied to candidate extended haplotypes of around 400 SNPs, as shown in Figures 5, 6 and 7, haplotype blocks are less informative. We considered various metrics to summarize haplotype block related data across the regions, such as median D', median multiallelic D', number of blocks, median block length, total possible variants, and percentage of informative SNPs, but were not satisfied with any single metric. In contrast, ExHap offers a single metric, the percentage of chromosomes that are congruent, to capture the commonalities across the region. Additionally, ExHap supports binary classification of individual chromosomes. Given a set of chromosomes, a consensus sequence is identified and individual chromosomes are classified as congruent or not. In comparison, the largest of the 8 haplotype blocks characterizing CEU chromosome 8 (Figure 6) contained 13 variants of 154 SNPs, while the second largest contained 13 variants of 55 SNPs.

Additionally, we compared selected features of ExHap to those of GERMLINE and Sweep. We also compared numeric results of the three programs for three chromosomal regions in family phased HapMap data. All three programs were designed to identify different features of haplotype matrices associated with different underlying biological phenomenon. However, the feature comparison in Table 2 and the numeric results in Table 3 provide additional insight into some of the tools available for the study of patterns of similarity in haplotype matrices.

There are limitations to our approach. One limitation is that the derived consensus sequence (and which chromosomes are considered "congruent") is dependent on the start site. An appropriate start site is particularly important for the long-range analysis. We determined start sites *a priori *(i.e., starting at *HLA-DRB1 *for the MHC region and continuing telomeric) or by inspecting larger allele identity plots (i.e., chromosome 2, 8 and 10 peaks). For example, in Figure 6, if looking for long-range congruence, a start site of 95 Mb would identify relatively low congruence, but a start site of 99 Mb (and going to about 101 Mb) would identify relatively high congruence. This is less of a problem for the rolling analysis as long as the length of the extended haplotype to be identified exceeds the length of the region of inspection. For example, using our parameters, we identify multiple extended haplotypes covering more than 250 SNPs, but probably miss extended haplotypes that are shorter than 250 SNPs. Our suggested strategy is to first use rolling analysis, with overlap and a moderate window (e.g. a 250 SNP window with a 50 SNP overlap), followed by long-range analysis once the appropriate range is identified. Another limitation of ExHap is that in a haplotype matrix with little congruence, only one chromosome in the matrix might be considered congruent (e.g., DR3-B7-A2 in Figure 3B).

Successful identification of extended haplotypes based on high density SNP data demands the accommodation of a low level of mismatches, whether due to experimental error (e.g., inaccurate genotype clustering) or point mutations. The ability of ExHap to accommodate experimental error might be particularly valuable in the analysis of data generated by genome-wide association studies, as the raw number of genotyping errors is more likely to increase when millions of SNPs are typed. Under our standard parameters, we require allele identity for 20 of 30 SNPs across each overlapping scoring window for a chromosome to be considered congruent. At first glance, this appears to be a rather weak definition of congruence. However, a chromosome must repeatedly and consistently match the emerging consensus string to be considered congruent. The actual allele identity within congruent chromosomes is typically very high (median 99.5% identity in our 132 T1DGC haplotype groups). We believe this is due to both the underlying haplotypes (the haplotypes either "match" or "don't") and to the use of a "look ahead" window that emphasizes longer haplotypes. That said, the matching rule is user-configurable. A more stringent matching rule (e.g., 28 of 30) might be appropriate if point mutations on a specific haplotype are implicated in disease. In general, parameter settings should be tuned to accommodate the nature of the data set (e.g. number of SNPs, SNP density) and the features under consideration.

The look-ahead window that emphasizes length is one of the important features of ExHap. The emphasis on length, coupled with allele identity plots, allows us to explore a set of chromosomes beyond the limits of a candidate or known extended haplotype (e.g. *HLADRB1 *to *HLA-B*), detecting patterns of congruence deterioration such as those illustrated in Figure 4. Another important feature is the ability to specify a starting point for congruence computation, such as *HLA-DRB1*. This feature was important in our study of the MHC as it allowed us to incorporate prior knowledge of haplotypes defined by HLA alleles alone.

ExHap was also designed to support the study of case and control chromosomes. The long-range linkage disequilibrium present on extended haplotypes confounds association analyses as specific risk alleles are difficult to localize given long-range linkage disequilibrium. However, long-range haplotypes are useful in identifying common complex disease associations related to a specific haplotype, where affected individuals share a long haplotype. By design, ExHap stratifies chromosomes into two classes, congruent and not congruent. Additionally, the program further tallies the results by case and control. The resulting data can be compared to, for example, investigate the difference in congruence between the two groups.

Furthermore, ExHap is a valuable tool for visualizing and quantifying patterns of similarity and differences in haplotype matrices. As such, it is useful for hypothesis generation. For example, by applying the tool to HapMap data, we identified regions that are highly congruent for both CEU and YRI, congruent for just CEU, or congruent for just YRI. Each of these categories is potentially interesting for different reasons. One might hypothesize that a region highly congruent for YRI but not CEU might be important for population health and survival in tropical Africa. A similar approach comparing congruence across disease cohorts might be of great value studying Immunochip Consortium data sets (nearly 200,000 SNPs, over 150,000 chips ordered in support of planned studies of diseases including rheumatoid arthritis, T1D, and multiple sclerosis) [35].

## Conclusions

In conclusion, we illustrate that congruence is informative for ranges of 250-2000 SNPs, both in the MHC and across the genome. A simple metric, the percentage of chromosomes that are congruent, easily highlights regions of interest for future research. In addition, binary classification of individual chromosomes as congruent or not congruent facilitates subsequent analysis. Furthermore, we can study individual chromosomes in the context of extended haplotypes using allele identity plots. This could prove important in disease applications where an individual's specific mutation is of interest. Thus, the ability to identify extended haplotypes and to inspect individual chromosomes using congruence is potentially valuable for both population and disease genetics.

## Abbreviations

AFBAC: Affected family-based control; CEU: CEPH HapMap population; EHH: Extended haplotype homozygosity; LCT: Lactase gene; MHC: Major histocompatibility complex; SNP: Single nucleotide polymorphism; T1DGC: Type 1 Diabetes Genetics Consortium; YRI: Yoruba HapMap population.

## Competing interests

JS is Founder and President of CytoAnalytics. EB, JJ, TB, PF, and GE declare they have no competing interests.

## Authors' contributions

Conceived and designed the experiments: EB, TB, and GE. Performed bioinformatic analysis: EB, JJ, and JS. Contributed to discussion: PF and GE. Wrote manuscript: EB and JS. All authors read and approved the final manuscript.

## Supplementary Material

Additional file 1 **ExHap User's Guide**. Click here for file

Additional file 2 **ExHap Software Distribution**. Click here for file

Additional file 3 **Table S2**. Congruence peaks may represent regions of extended haplotypes. This table includes chromosome, cohort, telomeric and centromeric positions identifying the limits of the peak region, the length of the region in megabases (Mb), and the length of the region in SNPs. For each cohort, the peak was defined as two or more contiguous ranges of 250 SNPs where congruence exceeded the 99^th ^percentile of congruence for that cohort (51.1% for CEU, 27.5% for YRI). Click here for file

Additional file 4 **Table S1**. Congruence of haplotype groups identified in the T1DGC data. Raw data for the bar chart in Figure 3A is shown, including percent of chromosomes that are congruent and the percent allele identity within those congruent chromosomes. Click here for file

Additional file 5 **Figure S1**. Congruence across the genome. These figures illustrate rolling short range congruence across chromosomes 1 through 22 for both CEU and YRI cohorts of the International HapMap Project. The horizontal reference lines (solid = CEH; dashed = YRI) show the 99th percentile of congruence (51.1% for CEU, 27.5% for YRI) for the respective cohorts. Overall, congruence was calculated for 27,652 overlapping ranges of 250 SNPs. Centromeres are indicated by "cen" at the starting position. Other loci of interest are indicated by the name [e.g. LCT or UNK (unknown)] and a source in which they are discussed (e.g. Sabeti2007). Click here for file

Additional file 6 **Table S3**. Genes contained in candidate extended haplotypes. The genes listed in the table are contained in candidate extended haplotypes of either the CEU cohort and/or the YRI cohort, according to data compiled in the UCSC Genome Browser database. Some genes are listed multiple times due to differences in start position and/or description. Click here for file

Additional file 7 **Table S4**. KEGG Pathways associated with genes in candidate extended haplotypes. The KEGG pathways listed in the table are associated with genes found in candidate extended haplotypes of either the CEU cohort and/or the YRI cohort, according to data compiled in the UCSC Genome Browser database. "Hits" is the number of genes associated with each pathway. Click here for file

Additional file 8 **Figure S2**. For chromosome 10, the maximum recombination rate was identified for each 250 SNP range for which congruence was calculated. These recombination rates are plotted against the corresponding congruence rates for the YRI cohort. Reference lines indicate the 90th percentile of the congruence rates (11%) and the 10th percentile of the recombination rates (17%). The points in the upper right hand quadrant represent those regions for which both congruence and recombination are relatively high and potentially warrant further investigation. Click here for file
