# Supplementary material for: Congruence as a measurement of extended haplotype structure across the genome
Source: J Transl Med. 2012 Feb 27;10:32. doi: 10.1186/1479-5876-10-32 (PMC3310717; doi:10.1186/1479-5876-10-32)
Supplement: Additional file 1 — ExHap User's Guide. [file 1479-5876-10-32-S1.PDF]

ExHap User's Guide

Version 1.0
December 2011
Janet Siebert
CytoAnalytics
www.cytoanalytics.com

DR3-B7-A2: 26 Chromosomes
1818 SNPs, 2.64 Mb
% Congruent=3.8, % Allele Identity=98.6

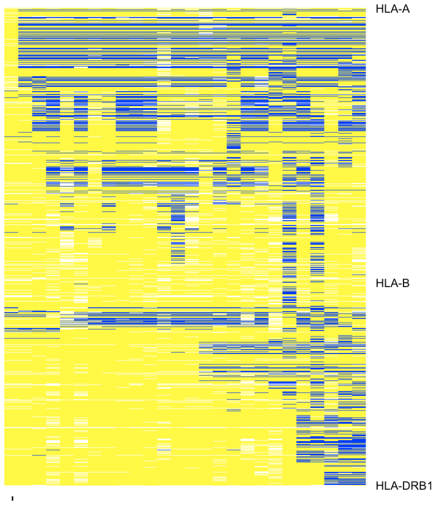

CytoAnalytics
Helping researchers find and tell the stories in their data

Table of Contents

1. Introduction ... 3
   1.1 Citing ExHap ... 4
   1.2 T1DGC and HapMap Examples ... 4
   1.3 Historical Notes ... 4
   1.4 Assumptions ... 4
   1.5 Files in the ExHap Software Distribution ... 4
2. Input Files ... 5
   2.1 Chromosome File ... 6
   2.2 Cohort Filter File ... 7
   2.3 Properties File ... 8
3. Usage ... 14
   3.1 Running Multiple Cohorts ... 15
   3.2 Running in "Rolling Congruence" Mode ... 16
   3.3 Filtering on Fields in the Chromosome File ... 17
4. Output Directories and Files ... 17
   4.1 Chromosome Statistics ... 18
   4.2 Heatmap File ... 18
   4.3 Allele Files ... 19
   4.4 Summary Metrics Written to Standard Output ... 22
   4.5 Allele Counts for Fisher's Exact Test ... 23
5. Detailed Description of the ExHap Algorithm ... 24
6. Impact of Parameter Settings and Handling of Ties ... 25
7. Limitations ... 26
8. Appendix: Configuration Properties in Alphabetical Order ... 26

1. Introduction

Historically, extended haplotypes have been defined using only a few data points, such as alleles for several HLA genes in the MHC. High-density SNP data, and the increasing affordability of whole genome SNP typing, creates the opportunity to define higher resolution extended haplotypes. This drives the need for new tools that are able to support quantification and visualization of extended haplotypes as defined by as many as 2000 SNPs. Confronted with high-density SNP data across the major histocompatibility complex (MHC) for 2,300 complete families, compiled by the Type 1 Diabetes Genetics Consortium (T1DGC), we developed software for studying extended haplotypes. The software, called ExHap (Extended Haplotype), uses a measurement we term congruence to identify and quantify long-range allele identity.

Given data representing a set of chromosomes as defined by a range of contiguous SNPs (hereafter referred to as “chromosomes”), ExHap derives a single consensus string that captures the commonalities among chromosomes. It also identifies congruent chromosomes (those chromosomes that have a high level of allele identity) and the percentage of allele identity between each chromosome and the consensus string. ExHap has two main parts: deriving a consensus string and filtering out chromosomes that do not match this string. The program (1) derives a consensus string by iteratively (a) positioning a derivation window of length W; (b) computing the most frequent string of length W within the derivation window; (c) appending the first N alleles of this most frequent substring to the consensus string; and (2) filters out chromosomes that do not match the emerging consensus string by (a) positioning the filter window; (b) within the filter window, checking SNPs from each chromosome for identity with the consensus string; and (c) removing non-congruent chromosomes from further consideration based on a matching rule for allele identity.

Summary output includes the consensus string, the number of chromosomes from each cohort (case and control) that are congruent to the consensus string, the number of chromosomes from each cohort that were evaluated, and the number of chromosomes flagged as unusable due to missing data. Corresponding percentages are also reported. Chromosome level output includes the chromosome identifier, the cohort, whether or not the chromosome was unusable due to missing data, whether or not the chromosome was congruent throughout the entire range of consideration, the congruence length, and the percentage of allele identity with the consensus string. Additionally, chromosome level output includes data preformatted for representation as a heatmap, showing whether or not each nucleotide from each chromosome matched the consensus string. This output is presorted from best matching to worst matching chromosomes, as illustrated in Figure 3B of Baschal et. al.(Section 1.1), thereby enabling easy chromosome-level inspection of conformance with known or putative extended haplotypes.

This algorithm is a heuristic, a rapid but approximate technique, for deriving a consensus string. A consensus string captures the commonalities among the inspected chromosomes within a given set. We cannot claim that it finds the best-possible consensus string, with the fewest number of mismatches in the largest number of chromosomes. However, it does identify a reasonable consensus string using a deterministic process. Run with the same parameters on the same set of data, it will always yield the same result, regardless of the order of the chromosomes in the data set. We are able to run the program

on over 9,000 chromosomes, inspecting over 2,000 SNPs, in less than an hour, on standard desktop personal computers. Smaller data sets can be evaluated in a few minutes.

1.1 Citing ExHap

If you use ExHap in published results, please cite the publication, *TODO: Insert reference, upon acceptance.*

1.2 T1DGC and HapMap Examples

ExHap was originally developed to identify conserved extended haplotypes across the MHC, in data compiled by the Type 1 Diabetes Genetics Consortium (T1DGC). In the T1DGC data, we commonly stratify chromosomes by haplotype groups based on MHC alleles (e.g. *HLA-DQB1*, *HLA-DRB1*, *HLA-B* and *HLA-A*) during the course of analysis. Additionally, we report results for case and control cohorts.

To show general utility of the program, we then applied it to data sets from the International HapMap Project. In analysis of the HapMap data, neither filtering by haplotype group nor reporting by case and control are relevant. Some of the examples below are drawn from the HapMap data set, and as such do not fully leverage the power of ExHap for filtering and for reporting by case and control.

1.3 Historical Notes

During the development of ExHap, our nomenclature evolved from "conserved" to "consensus" and "congruent." Thus, there are some output fields that still use the term "conserved" instead of "congruent." Additionally, since our original work was on Chromosome 6, a configuration property refers to "FlowCh6." While the name is currently hardcoded, this field can be populated with any appropriate value.

1.4 Assumptions

This User's Guide assumes access to a Unix or Unix-like command line interface (Linux, Cygwin, Mac OS X). While the program is written in Java and is thus platform independent, we only provide scripts and detailed instructions for such Unix-like environments. ExHap requires Java SE 6 or later.

1.5 Files in the ExHap Software Distribution

The ExHap software distribution is a zip file consisting of a jar file, several utility scripts, sample input files, and this User's Guide. These files are organized into a directory structure as follows:

bin

- **ExHap.jar** *(class files to run the program)*

input *(sample data and properties file)*

- **ceu.filter**

chr8_ceu_subset.csv *(a subset of HapMap chromosome 8, CEU cohort, ranging from chromosome position 94815225 to 115872654 (just over 9,500 SNPs), and approximately corresponding to Figure 5 in Baschal et al. as cited above).*
consensus.properties

output *(directory structure to support 3 types of output)*

scripts
runExHap.sh
runExHapRolling.sh
wrapExHap.sh

src *(Java source files)*

2. Input Files

The program uses the following files as input. Generally, file names and certain features of the files are specified in a properties file. The names of the corresponding properties are noted.

File	Description	Relevant Configuration Property Names
Chromosome file	Required. Comma delimited file, containing chromosome data. Must have at least 2 header rows. One header row contains SNP name and the other column headers. The second header row contains chromosome position of that SNP. The row number of each of these rows is a configuration parameter in the properties file. See the documentation below for additional details on this file.	`fileName`<br>`headerRow`<br>`majorAlleleRow`<br>`positionRow`<br>`caseFieldName`<br>`caseDesignator`
Cohort filter file	Optional. A comma delimited file containing analid, haplotype cohort (e.g. 3.8.1), any other field, and Ch6 flow. The file must have at least 4 columns, and they must be in the order mentioned above. Specific column headers are not required. See example below.	`filterFile`
Properties file	Required. This file specifies the various program configuration parameters. Details are provided in a separate section.	`Name of file is passed to the program`

Annotations file	Optional. This file specifies text strings to be output adjacent to specific SNPs. Can be used to highlight specific genes.	`annotationsFile`

2.1 Chromosome File

The table below shows an extract of a chromosome file with 9 header rows and 3 data rows. Only a few of the SNP data columns are shown. There is no programmed limit on the number of columns that can precede the SNP data.

1						*Major_Allele*	*T*	*A*	*A*
2						*Minor_Allele*	*G*	*G*	*G*
3						*Redundant*	*0*	*0*	*0*
4						*Success*	*1*	*1*	*1*
5						*B36_ref_locus*		*OR12D3*	*OR12D3*
6						*Major_AF*	*0.514646*	*0.787236808*	*0.9596366*
7						*Minor_AF*	*0.485354*	*0.212763192*	*0.0403634*
8						*B36_Position*	*29299390*	*29448986*	*29450215*
**9**	**analid**	**FlowCh6**	**ASP_Hap_Status**	**hla_drb_4digit**	**hla_dqa**	**hla_dqb_4digit**	**rs714470**	**rs7772982**	**rs9380122**
10	10060001	A	2	401	301	302	4	1	1
11	10060001	B	0	701	201	201	3	1	3
12	10060002	C	2	301	501	201	3	3	1

The chromosome file is a comma-delimited file containing the phased chromosome data and a variety of supplemental information that is useful for downstream analysis. For example, ASP_Hap_Status (ASP=affected sibling-pair) contains case/control founder chromosome assignment. Its derivation is discussed in Baschal *et. al* as referenced above. In this context, all of the chromosomes under study are founder chromosomes, but that is not a requirement of the program. There is no requirement that there be two rows of chromosome data for each person. This might be the case if you were analyzing only chromosomes that had a particular allele for a particular SNP or gene.

The file consists of 3 main parts:

- Main header row (shown in bold, and row 9 in this case)
- SNP header rows (shown in italics)
- Chromosome and donor data

The main header row (row 9 in this example) contains labels for each column. Once the SNP data begins, past the extra descriptive columns, the column heading in row 9 is the SNP name. Above row 9 there are SNP header rows that provide additional information about the SNP, including major and minor allele and chromosome position. SNP name, chromosome position, and major allele are all used in the program. The row numbers on which SNP name, chromosome position, and major allele appear are configurable in the properties file. If your data does not have a major allele row and you have no missing alleles in your data set (as in the HapMap data), you can set the configuration property

majorAlleleRow to point to the position row. If the consensus string as derived contains an allele that is unknown/unphased, the unknown/unphased SNP is replaced with the appropriate value in the majorAlleleRow.

The program assumes that there will be columns labeled "analid" and "FlowCh6". It uses these two columns to create a chromosome id consisting of the analid and the flow designator, e.g. 10060001_A and 10060001_B. If your data doesn't have the logical equivalent of FlowCh6, a dummy column containing a constant value can be added. The program also assumes that there will be a column that defines Case and Control. The name of this column is specified in the properties file. Also, the value that represents case is specified in the properties file. If your data does not have a case and control designator, we suggest using the FlowCh6 as the caseFieldName. Keep in mind that results reported for case and control cohorts may not be meaningful when there are not clear case and control cohorts in the data.

Alleles must be represented as 1, 2, 3, or 4 (corresponding to A, C, G or T). Unknown or unphased alleles must be represented as ? or N.

2.2 Cohort Filter File

The cohort filter file can be used to identfy a subset of chromosomes for analysis. It has 4 columns. The values in the first and last columns must match the values in the columns labeled "analid" and "FlowCh6" in the chromosome file. The second column indicates the group or cohort to be analyzed. These are intended to be different groupings than "case" and "control." The value to match is passed to ExHap on the command line. The third column is not used by ExHap. The cohort filter file must have a header row, but the values of the header row are irrelevant.

```
analid,Group,analid flow,FlowCh6
27268301,1.14.3,27268301B,B
40528302,1.14.3,40528302D,D
40862901,1.14.3,40862901B,B
43155701,1.14.3,43155701A,A
43716302,1.14.3,43716302C,C
```

2.3 Properties File

A sample properties file is shown below:

```
fileName=Data/ExHap/out_4thpass_old/subsets/allFounder.H.csv
filterFile=Data/ExHap/haplogroups/haplogroup.csv
delim=,
debug=true

#note:  row numbers are 1-based
headerRow=9
majorAlleleRow=1
positionRow=8


#DRB1 to HLA-A
telomericLocus=rs1137078
centromericLocus=rs1059614
unphased=x
lengthToInspect=100
inspectionStep=10

matchLength=30
matchStep=5
mismatchLimit=10

scoreFrequency=10

caseFieldName=ASP_Hap_Status
caseDesignator=2

#parameters for failed chromosomes
contiguousThold=20
pctUnphasedThold=20
chromosomeStatsParentDir=Data/ExHap/haplo_stats/
chromoTag=Conserved To HLA_A
heatmapOutputDir=Data/ExHap/haplo_heatmaps/

outputAnnotations=true
annotationsFile=Data/ExHap/properties/annotations.csv

outputAlleles=true
alleleOutputDir=Data/ExHap/haplo_allele/
```

To configure ExHap, specify the properties described below. This table is organized into functional categories. There is another version of this table in the Appendix that lists parameters alphabetically. Not all properties documented herein are included in the sample properties file.

Any of these properties can be specified on the command line, using the –Dcyto.<propertyName>=<value> suntax. See the documentation on rolling congruence for an example.

**Description**	**Property Name**
Input files and associated parameters	
The fully qualified path name (e.g. directory and file name) of the chromosome file	`filename`
The fully qualified path name (e.g. directory and file name) of the cohort filter file, to be used to filter the chromosome file based on cohort. Cohort refers to a subset of chromosomes (e.g. a haplotype group) that can be selected for analysis from a larger set of chromosomes.	`filterFile`
A true/false value specifying whether the subset of chromosomes to be analyzed are specified by a list of fields embedded in the chromosome file	`filterByFields`
A comma delimited list of fields in the chromosome file to be used to define the filter (if `filterByFields=true`), e.g. filterFields=hla_drb_4digit,hla_dqa,hla_dqb_4digit	`filterFields`
A comma delimited list of values in the chromosome file to match (if `filterByFields=true`), e.g. filterValues=1501,102,602	`filterValues`
The delimiter of the chromosome file and filter file (only comma(",") has been thoroughly tested)	`Delim`
The row number of the chromosome file on which the SNP names appear. Row numbers start at 1.	`headerRow`
The row number of the chromosome file on which the Major Allele information appears. Row numbers start at 1. If the most common allele for a particular SNP is unphased or unknown, ExHap uses the major allele as the consensus value.	`majorAlleleRow`
The row number of the chromosome file on which the chromosome position appears. This value will be output as part of the heatmap files.	`positionRow`

Description	Property Name
The starting and ending SNPs of the analysis region. ExHap assumes that the SNP data is arranged in order from lowest position (telomere) to highest position (centromere), moving from left to right. ExHap calculates the consensus from the centromere (rightmost SNP) to the telomere (leftmost SNP). The telomeric and centromeric terminology is based on the original application to the MHC, on the short arm of chromosome 6.	`telomericLocus`<br>`centromericLocus`
A value that indicates how the SNPs for telomericLocus and centromericLocus are specified. Options are:<br>byName<br>stringPosition<br>byBasePosition<br><br>byName means that the values will be something like rs389419<br>byBasePosition means the value with be something like 29468162<br><br>For either byName or byBasePosition, the values must be found in the appropriate header row of the chromsome file. Additionally, the value for the centromericLocus must be to the right of the value for the telomericLocus.<br><br>stringPosition refers to the column number in the chromosome file. This is useful for rolling congruence calculations, where ExHap is executed in a loop with telomericLocus and centromericLocus passed as properties on the command line.	`snpIdentification`
The character to be used for textual representation of unphased/unknown alleles in the output. Unknown or unphased alleles in the chromsome input file must be represented as ? or N.	`unphased`
The label in the header row of the chromosome file that specifies the case-control field	`caseFieldName`
The value in the field in the chromosome file specified by `caseFieldName` that represents "case." All other values will be counted as control.	`caseDesignator`

**Description**	**Property Name**
Consensus determination	
Starting at the most centromeric of the SNPs, ExHap looks at W (lengthToInspect) SNPs, and determines the most common pattern of SNPs for that W-length sequence. ExHap then records as "consensus" the first N (inspectionStep) SNPs from the most common pattern. Then, ExHap increments its inspection by N SNPs, and repeats the process. See Section 5 for more details.	
The number of SNPs to inspect to compute a common pattern (W, as described in Section 5)	`lengthToInspect`
The number of SNPs to record in the consensus pattern, and to step forward by (N, as described in Section 5)	`inspectionStep`
When ExHap scores a SNP as matching the consensus string or not, it looks at a certain number of SNPs (M, matchLength) and counts mismatches from the consensus within a specified range. If the number of mismatches exceeds a specified limit (L, mismatchLimit), the chromosome is marked as not conserved. ExHap then moves forward by a certain number of SNPs (matchStep), and repeats the process. See Section 5 for more details.	
The number of SNPs to consider when determining whether or not a chromosome is congruent with the consensus string (M, as described in Section 5).	`matchLength`
The maximum number of mismatches allowed within the specified number of SNPs (L, as described in Section 5).	`mismatchLimit`
The number of positions to move forward before repeating the filtering out of non-matching chromosomes.	`matchStep`
ExHap scores the remaining non-failed conserved chromosomes to see if they are still conserved based on this parameter. For example, scoreFrequency=1 scores the chromosomes every time they are inspected; scoreFrequency=5 scores the chromosomes every 5th inspection.	`scoreFrequency`
Prior to computing a consensus sequence, ExHap marks some chromosomes as failed, or not of sufficient quality to be included in the analysis. A chromosome is excluded for one of two reasons: (1) the number of contiguous unphased SNPs exceeds some limit on absolute count, or (2) the number of unphased/unknown chromosomes across the entire region of consideration (e.g. *HLA-DRB1* to *HLA-A*) exceeds some percentage threshold.	
The maximum number of contiguous unphased/unknown chromosomes allowed.	`contiguousThold`
The percentage of unphased/unknown chromosomes allowed across the entire region.	`pctUnphasedThold`

Description	Property Name
Output location and configurations	
A true/false value indicating whether or not verbose output should be written to the output stream. This stream can be routed to a file.	`Debug`
The directory to which chromosome statistics are written.	`chromosomeStatsParentDir`
The phrase that will be part of the chromosome statistics file, and the chromosome statistics file name, e.g. "Conserved To A". Spaces in the phrase will be removed when the file name is created.	`chromoTag`
The directory to which the heatmap files should be written. Note that the naming convention for the files is <cohort>.heatmap.txt, e.g. 3.8.1.heatmap.txt	`heatmapOutputDir`
A true/false value indicating whether or not annotations should be included in the heatmap and allele files	`outputAnnotations`
The fully qualified path name for a comma delimited file containing SNP annotations. The structure of the file is <snp_name>,<text annotation>, as shown below:<br><br>`rs1059614,drb1`<br>`rs9266247,hla_b`<br>`rs1137078,hla_a`<br>`rs995185,UBD`<br><br>The order of the annotations does not need to correspond to the order of the SNPs in the chromosome file. However, only one line is allowed per SNP.	`annotationsFile`
A true/false value indicating whether or not allele files should be output.	`outputAlleles`
The directory to which the allele output file should be written. The file names are <chromoTag>.<cohort>.summary.txt and <chromoTag>.<cohort>.alleles.txt<br><br>The directory name should end with a /	`alleleOutputDir`

Description	Property Name
A true/false value controlling the presence of a custom row label in the heatmap and allele files. This label can be created from any combination and any order of<br>numUnphased, snp, position, and defaultMajorAllele, e.g.<br><br>outputCustomRowLabels=true<br>labelParams=snp,position,defaultMajorAllele<br>labelDelim=_	`outputCustomRowLabels`
A comma separated list of parameters to include in the custom row label (used when `outputCustomRowLabel=true`), e.g. snp,position,defaultMajorAllele	`labelParams`
The character used to separate the values of the `labelParams`, e.g. "_"	`labelDelim`
Using a previously derived consensus sequence	
A true/false value controlling the use of a consensus sequence that has been previously derived. This feature supports computation of consensus on one cohort, and then using that consensus sequence to calculate congruence metrics for a different cohort.	`readConsensusFromFile`
The path to and name of an allele summary file that contains a previously computed consensus sequence, used when `readConsensusFromFile=true`	`consensusFileName`

3. Usage

All usage examples assume that the working directory is the scripts directory of the distribution.

The usage of ExHap is as follows:

```
./runExHap.sh <properties_file> <cohort>
```

Given the files in the ExHap distribution, the following command will run the program. Since there are no cohorts within a given HapMap population, the second argument (0) refers to a placeholder value in the filter file:

```
./runExHap.sh ../input/consensus.properties 0
```

To compute congruence on all data in a chromosome file, specify "all" as the cohort, e.g.

```
./runExHap.sh ../input/consensus.properties all
```

Output can be redirected to a file with the following command:

```
./runExHap.sh consensus.properties 0 > myTest.out
```

A variety of summary metrics are available in the output by grepping for the phrase "Summary", e.g.

```
grep Summary myTest.out
```

Representative output:

```
,SummaryConsensus,0,CAAAGAGTTATGAG etc.)
,SummaryCaseControl,0,Case Conserved,Case Non-conserved,Control Conserved,Control
Non-conserved,0,88,1,87
,SummaryStats,0,Inspect,100,Record,5,Mismatch Limit,10,Mismatch Length,30,Match
Step,10,Failed,0,CountConserved,1,CountCanUse,176,Pct,0.6,Case Conserved,0,Case
Total,88,Case % Conserved,0%,Control Conserved,1,Control Total,88,Control %
Conserved,1.1%
SimpleSummary,10,10 to 500,teloPosition,94841844,centroPosition,95831847,
BasePairRange,990003,CountConserved,1,CountCanUse,176,Pct,0.6,ConservedPctIdentity,
95.9
```

The details on interpreting this summary output are provided in Section 4.

3.1 Running Multiple Cohorts

The script, wrapExHap.sh, provides a mechanism for running multiple cohorts with one command.

Usage of the script is as follows:

```
./wrapExHap.sh <file containing list of cohorts>
```

For example:

```
./wrapExHap.sh cohorts_long
```

In this case, the contents of cohorts_long might be:

```
1.35.3
15.18.25
3.18.30
3.8.1
4.15.2
4.44.2
7.44.29
8.39.24
8.40.2
all
```

The contents of the script are:

```
for i in `cat $1`
do
./runExHap.sh ../input/consensus.properties $i >  ../output/$i.out
done
```

The two sections in bold specify the location and name of the properties file, and the location and name of the output directory.

The combination of the for loop and the cat command have the effect of taking each cohort listed in the file name specified on the command line, and executing runExHap.sh for each cohort. Output is directed to the specified directory and a file named <cohort>.out

Summary data from all runs can be viewed by executing the following command in the output directory:

```
grep Summary *.out
```

Representative output is shown below:

```
1.35.2.out:SummaryStats, Inspect,100,10, Mismatch,10,30, step,5,
Failed,2, countConserved,1,countCanUse,19, pct,5.3, CaseControl,
case,1,8, control,0,11
1.35.3.out:SummaryStats, Inspect,100,10, Mismatch,10,30, step,5,
Failed,7, countConserved,3,countCanUse,61, pct,4.9, CaseControl,
case,1,27, control,2,34
```

```
15.18.25.out:SummaryStats, Inspect,100,10, Mismatch,10,30, step,5,
Failed,1, countConserved,14,countCanUse,17, pct,82.4, CaseControl,
case,0,0, control,14,17
3.18.30.out:SummaryStats, Inspect,100,10, Mismatch,10,30, step,5,
Failed,8, countConserved,141,countCanUse,151, pct,93.4, CaseControl,
case,124,133, control,17,18
```

3.2 Running in "Rolling Congruence" Mode

The script, runExHapRolling.sh provides an example of running ExHap in rolling congruence mode. Essentially, the script executes ExHap multiple times, each time computing congruence for the SNPs ranging in position from the variables i to j. For the HapMap data, we inspected 250 SNPs per iteration, incrementing the start position by 50 for each iteration. By using the java –D option to specify system properties, we are able to pass key parameters from the script to the program. Note that all of the properties are specified as cyto.<parameterName>, e.g. cyto.telomericLocus.

Representative usage is:

```
./runExHapRolling.sh ../input/chr8_ceu_subset.csv ../input/ceu.filter 4000
```

Arguments are chromosomeFile, filterFile, and the upper bound of the string position of the telomeric SNP for which you want to compute congruence. The upper bound of the string position of the centromeric SNP is computed by adding some number (in this case, 249; for a range of 250 SNPs) to the telomeric position, which is also the control variable of the while loop. In this case, the rolling window is incremented by 50 SNPs every interation.

The contents of the script are:

```
#! /bin/bash
i=2

while((i < $3 ))
do
    echo $i
    j=$i
    ((j += 249))
    java -cp "../bin/ExHap.jar" -Dcyto.fileName=$1 -
Dcyto.filterFile="$2" -Dcyto.telomericLocus=$i -
Dcyto.centromericLocus=$j -Dcyto.chromoTag=$i
com.cytoanalytics.filetools.Consensus ../input/consensus.properties 0
    ((i +=50))
done
```

3.3 Filtering on Fields in the Chromosome File

If the chromosome file contains fields that are of interest in identifying a subset of chromosomes for analysis, these fields can be used to filter the chromosome file, eliminating the need to have an external filtering file. Examples include HLA alleles, alleles of other genes that have been phased, or even certain SNPs themselves. To use this feature, set properties per the examples below. The labels used for the `filterFields` are those appearing in the `headerRow` of the chromosome file. There should be a one-to-one correspondence between the fields listed in `filterFields` and the values listed in `filterValues`. The lists must be comma-delimited. "Not equal" functionality can be employed by prefacing the filter value with the negation operator, "!".

```
#filtering on a single field
filterByFields=true
filterFields=hla_b_4digit
filterValues=1402

#filtering on a set of fields
filterByFields=true
filterFields=hla_drb_4digit,hla_dqb_4digit,hla_b_4digit,hla_a_4digit
filterValues=404,302,1501,201

#filtering on a set of fields, with negation (!4)
filterByFields=true
filterFields=hla_drb_2digit,hla_b_4digit,hla_a_4digit
filterValues=!4,1501,201
```

4. Output Directories and Files

The program places output in the following directories, which are specified in the properties file. The directories will be created if they do not exist

Directory Description	Property
Output directory for chromosome statistical data. Output files are created in subdirectories corresponding to program configuration information.	`chromosomeStatsParentDir`
Output directory for heatmap files, designed to be read by MEV, available from http://www.tm4.org/mev.html.	`heatmapOutputDir`
Output directory for allele/haplotype files, corresponding in structure to the heatmap file.	`alleleOutputDir`

4.1 Chromosome Statistics

Chromosome statistics are recorded in a directory structure that allows unambiguous identification of the rules that were used to generate the data. Several example directories are shown below:

```
Inspect_100_Store_10_MismatchLimit_10_Of_30_Step_5
Inspect_20_Store_5_MismatchLimit_10_Of_30_Step_5
Inspect_5_Store_4_MismatchLimit_2_Of_5_Step_1
```

Within a directory, scores are written to .csv files specified by <cohort>.<chomoTag>.ChromoScore.csv. Any spaces present in the chromoTag (as specified in the properties file are) are removed for the creation of the file name. Representative file names are below:

```
1.35.2.ConservedToHLA_A.ChromoScore.csv
3.18.30.ConservedToHLA_A.ChromoScore.csv
4.44.2.ConservedToHLA_A.ChromoScore.csv
1.35.3.ConservedToHLA_A.ChromoScore.csv
3.8.1.ConservedToHLA_A.ChromoScore.csv
7.44.29.ConservedToHLA_A.ChromoScore.csv
15.18.25.ConservedToHLA_A.ChromoScore.csv
4.15.2.ConservedToHLA_A.ChromoScore.csv
8.40.2.ConservedToHLA_A.ChromoScore.csv
```

Data within each file consists of the chromosome identifier, the cohort, the configuration rule, the tag, information on whether or not the chromosome is usable (e.g. did not fail), information on whether or not the chromosome is conserved, and the length of the conservation. This somewhat verbose output is designed to provide all needed information on each chromosome, plus traceability to the program configuration that computed the statistics.

```
23640002_D,8.40.2,Inspect_100_Store_10_MismatchLimit_10_Of_30_Step_5,Conserved To HLA_A,CanUse,true,Conserved,true,LengthConserved,1818
20510801_B,8.40.2,Inspect_100_Store_10_MismatchLimit_10_Of_30_Step_5,Conserved To HLA_A,CanUse,true,Conserved,true,LengthConserved,1818
40586601_B,8.40.2,Inspect_100_Store_10_MismatchLimit_10_Of_30_Step_5,Conserved To HLA_A,CanUse,true,Conserved,true,LengthConserved,1818
45572901_B,8.40.2,Inspect_100_Store_10_MismatchLimit_10_Of_30_Step_5,Conserved To HLA_A,CanUse,true,Conserved,true,LengthConserved,1818
47595502_D,8.40.2,Inspect_100_Store_10_MismatchLimit_10_Of_30_Step_5,Conserved To HLA_A,CanUse,true,Conserved,true,LengthConserved,1818
55144601_B,8.40.2,Inspect_100_Store_10_MismatchLimit_10_Of_30_Step_5,Conserved To HLA_A,CanUse,true,Conserved,true,LengthConserved,1818
52970502_D,8.40.2,Inspect_100_Store_10_MismatchLimit_10_Of_30_Step_5,Conserved To HLA_A,CanUse,true,Conserved,false,LengthConserved,570
50122702_C,8.40.2,Inspect_100_Store_10_MismatchLimit_10_Of_30_Step_5,Conserved To HLA_A,CanUse,true,Conserved,false,LengthConserved,200
50623701_B,8.40.2,Inspect_100_Store_10_MismatchLimit_10_Of_30_Step_5,Conserved To HLA_A,CanUse,true,Conserved,false,LengthConserved,180
```

4.2 Heatmap File

The heatmap file is preformatted for visualization with TM4 MEV software (http://www.tm4.org/mev/). We have also used the heatmap output with the R package heatmap2.

An extract of the file is below. Column names have been rotated to conserve space in this document.

An explanation of each column follows. The ConsAllele is the allele that is determined to be part of the consensus sequence. The posit value is the ordinal position of the SNP in the range of inspection. chPosit is the position on the chromosome, as specified in the chromosome file. NumUnphased is the number of chromosomes for which that SNP is unknown or unphased. Annotation is the custom annotation (if any) associated with that SNP position. Custom (not shown) is the composite tag that is

made up of any combination of numUnphased, snp, position, and defaultMajorAllele, as specified in the properties file. It can be useful when viewing the data as a heatmap in MEV. The chromosome label (e.g. NA19095_A_A_450) is a composite label for chromosome indicating the id, case or control, and the number of SNPs for which the chromosome is congruent. The values of -3, 0, and 3 correspond to "does not match the consensus allele", "unknown/unphased", and "does match the consensus allele." These values correspond to the default values expected by MEV.

NAME	ConsAllele	MajAllele	posit	chPosit	NumUnphased	Annotation	NA19095_A_A_450	NA18868_B_0_450	NA18924_B_0_450
rs7924066	G	1	450	103022099	0		-3	-3	-3
rs12569599	G	1	449	103023116	0		3	3	-3
rs11190901	C	1	448	103029254	0		3	-3	3
rs606034	G	1	447	103036358	0		3	3	3
rs644633	A	1	446	103040561	0		3	3	3
rs614375	C	1	445	103044972	0		3	3	3
rs11190912	G	1	444	103046293	0		3	3	3
rs11190913	G	1	443	103049028	0		3	3	3
rs616983	C	1	442	103049890	0		-3	3	3
rs927351	A	1	441	103052302	0		3	3	3
rs11597176	G	1	440	103053070	0		3	3	3

4.3 Allele Files

Allele files are written to the directory specified by the property, alleleOutputDir. There is both a summary file and a detailed allele file. The naming convention for the summary file is <cohort>.<tag>.summary.txt while the naming convention for the detailed allele file is <cohort>.<tag>.alleles.txt

An extract of the summary file is below. The format is similar to that of the heatmap file. Column names have been rotated to conserve space in this document.

An explanation of each column follows. The ConsAllele is the allele which is determined to be part of the consensus sequence. The posit value is the ordinal position of the SNP in the range of inspection. chPosit is the position on the chromosome, as specified in the chromosome file. NumUnphased is the number of chromosomes for which that SNP is unknown or unphased. Annotation is the custom annotation (if any) associated with that SNP position. Custom is the composite tag that is made up of any combination of numUnphased, snp, position, and defaultMajorAllele, as specified in the properties file.

`<cohort>.<tag>.summary.txt`

NAME	ConsAllele	MajAllele	posit	chPosit	NumUnphased	Annotation	Custom
rs9266247	G	G	765	31433735	185	hla_b	rs9266247_31433735_G
rs2523590	A	A	764	31435043	77		rs2523590_31435043_A
rs2523589	T	G	763	31435313	76		rs2523589_31435313_G
rs2523586	T	T	762	31435414	63		rs2523586_31435414_T
rs1811197	C	C	761	31435639	126		rs1811197_31435639_C
rs2523578	A	A	760	31436521	281		rs2523578_31436521_A
rs2523567	G	G	759	31437994	37		rs2523567_31437994_G
rs2596548	G	G	758	31438525	21		rs2596548_31438525_G
rs2523554	A	A	757	31439808	47		rs2523554_31439808_A

The data included in the allele file is the same as that in the summary file, with an additional column for each chromosome. The alleles, expressed as A, C, G, or T, are shown for each SNP.

10103601_A_2_765 is a sample of a composite label for a chromosome indicating the id, case or control, and the number of SNPs for which the chromosome is congruent.

`<cohort>.<tag>.alleles.txt`

NAME	ConsAllele	MajAllele	posit	chPosit	NumUnphased	Annotation	Custom	10103601_A_2_765	10340401_A_2_765	10393301_A_2_765	10632902_D_0_765
rs9266247	G	G	765	31433735	185	hla_b	rs9266247_31433735_G	x	x	x	x
rs2523590	A	A	764	31435043	77		rs2523590_31435043_A	A	A	x	A
rs2523589	T	G	763	31435313	76		rs2523589_31435313_G	T	T	T	T
rs2523586	T	T	762	31435414	63		rs2523586_31435414_T	T	T	T	T
rs1811197	C	C	761	31435639	126		rs1811197_31435639_C	x	x	x	x
rs2523578	A	A	760	31436521	281		rs2523578_31436521_A	A	A	A	A
rs2523567	G	G	759	31437994	37		rs2523567_31437994_G	G	G	G	G
rs2596548	G	G	758	31438525	21		rs2596548_31438525_G	G	G	G	G
rs2523554	A	A	757	31439808	47		rs2523554_31439808_A	A	A	A	A

4.4 Summary Metrics Written to Standard Output

Among the output included in the standard output stream are a variety of summary statistics. Grepping for "Summary" will yield lines like the following. Line numbers have been added for ease of reference.

```
1. ,SummaryConsensus,0,CAAAGAGTTATGAG etc.)
2. ,SummaryCaseControl,0,Case Conserved,Case Non-conserved,Control
   Conserved,Control Non-conserved,0,88,1,87
3. ,SummaryStats,0,Inspect,100,Record,5,Mismatch Limit,10,Mismatch
   Length,30,Match
   Step,10,Failed,0,CountConserved,1,CountCanUse,176,Pct,0.6,Case
   Conserved,0,Case Total,88,Case % Conserved,0%,Control Conserved,1,Control
   Total,88,Control % Conserved,1.1%
4. SimpleSummary,10,10 to
   500,teloPosition,94841844,centroPosition,95831847,BasePairRange,990003,CountC
   onserved,1,CountCanUse,176,Pct,0.6,ConservedPctIdentity,95.9
```

Interpretation is as follows.
Lines 1-3 include the cohort immediately after the line label. This is useful for aggregating data across multiple cohorts.
Line 1 is the final consensus sequence.
Line 2 provides four numbers, which are Case Conserved, Case Non-conserved, Control Conserved, and Control Non-conserved respectively.
Line 3 provides key configuration properties and a variety of metrics. Beginning with "Inspect," the property or metric is followed by the resulting value. Additional details are in the table below.

**Parameter/Metric**	**Value From Output**	**Explanation**
Inspect	100	See properties
Record	5	See properties
Mismatch Limit	10	See properties
Mismatch Length	30	See properties
Match Step	10	See properties
Failed	0	Number of chromosomes failing quality rules
CountConserved	1	Total number conserved
CountCanUse	176	Total number passing quality rules
Pct	0.6	Congruent or conserved chromsomes as a percent of usable/passing chromosomes
Case Conserved	0	Number of case chromosomes conserved
Case Total	88	Total number of usable case chromosomes
Case % Conserved	0%	Congruent or conserved case chromosomes as a percent of usable/passing case chromosomes
Control Conserved	1	As for case
Control Total	88	As for case
Control % Conserved	1.1%	As for case

Line 4 is a quick summary intended to support reporting of rolling congruence. Metrics are as per line 3, with the addition of "ConservedPctIdentity." Percent Identity is calculated first for an individual chromosome and is the percentage of known/phased alleles that match the consensus sequence. This

number is then averaged across all of the congruent chromosomes and reported as "ConservedPctIdentity."

4.5 Allele Counts for Fisher's Exact Test

ExHap also generates output preformatted for bulk calculation of Fisher's Exact Test. This information can be extracted from the standard output by grepping for the phrase "AlleleInfoForFisher". Representative comma-delimited output is shown below. The ObservedAlleles column lists the alleles that appeared in the analyzed data for the particular SNP (e.g. C or AG). The alleles are listed in alphabetical order. The columns, countCaseFirst and countCtlFirst, show the number of times the first allele (e.g. A, in the case of observed alleles AG) appears in the case chromosomes and the control chromosomes respectively.

```
AlleleInfoForFisher,SNP,Position,numA,numC,numG,numT,ObservedAlleles,
countCaseFirst,countCtlFirst,countCaseSecond,countControlSecond
AlleleInfoForFisher,rs3134021,94841844,0,176,0,0,C,88,88
AlleleInfoForFisher,rs3134024,94849126,49,0,127,0,AG,27,22,61,66
AlleleInfoForFisher,rs3097418,94851907,49,0,127,0,AG,27,22,61,66
AlleleInfoForFisher,rs4735238,94858857,103,0,73,0,AG,55,48,33,40
AlleleInfoForFisher,rs35793208,94862063,2,0,174,0,AG,2,0,86,88
```

The representative output is also illustrated in the table below for readability.

SNP	Position	numA	numC	numG	numT	ObservedAlleles	countCaseFirst	countCtlFirst	countCaseSecond	countControlSecond
rs3134021	94841844	0	176	0	0	C	88	88		
rs3134024	94849126	49	0	127	0	AG	27	22	61	66
rs3097418	94851907	49	0	127	0	AG	27	22	61	66
rs4735238	94858857	103	0	73	0	AG	55	48	33	40
rs35793208	94862063	2	0	174	0	AG	2	0	86	88

5. Detailed Description of the ExHap Algorithm

ExHap inspects SNP data for a set of chromosomes over a user-specified range of contiguous SNPs and derives a single consensus string that is similar to a large number of the chromosomes in the set. It also identifies congruent chromosomes and computes the percentage of chromosomes that are congruent to the consensus chromosome. A consensus string is a sequence of the most frequently occurring SNP substrings. With default settings, a congruent chromosome must match the consensus string for at least 20 out of 30 contiguous SNPs evaluated at multiple overlapping windows across the region of interest. A chromosome is labeled as not congruent and eliminated from future consideration the first time it fails to meet the matching criteria. At a high level, the algorithm operates as follows:

1. **Select chromosomes of interest across a SNP range of interest from a given haplotype matrix**. A haplotype matrix consists of multiple chromosomes and SNP nucleotides arranged in rows and columns. The user can specify a subset of chromosomes (e.g., those matching a particular HLA haplotype group) and a range of SNPs (e.g., *HLA-DRB1* to *HLA-A*).
2. **Eliminate chromosomes that are unusable due to missing data.** Chromosomes are flagged as unusable if they have a large percentage (e.g., 20%) of missing SNPs or have too many contiguous missing SNPs (e.g., 20). Both of these parameters are user-configurable.
3. **Iteratively, derive a consensus string, as illustrated in Figure 2.**
    a. **Position the derivation window**. Starting at the centromeric end of the range of SNPs, a specified number W of contiguous SNPs is selected from each chromosome in the set. We refer to this collection of sequences of length W as the derivation window.
    b. **Compute the most frequent string of length W within the derivation window.** The number of instances of each unique sequence of W SNPs is computed and the most frequent sequence is identified. For example, if W=9, we might find the most frequent sequence to be AAACCCTTT. In the event of a tie, the first sequence, in alphabetical order, is selected. See section 6 for a more details on ties.
    c. **Add the prefix or *substring*, whose length is a specified number N, of the most frequent string as part of the consensus sequence.** Continuing our example, if N=3, we would record the subsequence AAA.
4. **Filter out non-matching chromosomes.**
    a. **Position filter window.** Starting at the centromeric end of the region of interest, position the filter window of width M. In each filtering iteration, slide the window by a user-specified offset.
    b. **Check SNPs from each chromosome for identity with consensus string.** For each chromosome, compare it to the emerging consensus. For a filter window of size M, count the number of nucleotides in the chromosome that do not match the consensus. If this number exceeds the mismatch limit L, mark the chromosome as non-congruent. Both M and L are user-configurable. We commonly use M=30 and L=10, thereby forcing congruent chromosomes to have at least two-thirds of their nucleotides in agreement with the consensus for multiple overlapping ranges across the region under investigation. Unknown/unphased SNPs are not counted as mismatched.
    c. **Remove non-congruent chromosomes from congruence calculations**. Flag the non-congruent chromosomes as such. Record the number of SNPs for which they maintained congruence prior to becoming non-congruent. This measurement is referred to as the

congruence length. Chromosomes that are congruent across the entire range of interest have a congruence length equal to the number of SNPs in the range.

5. **Repeat steps 3 and 4 until the entire range has been inspected**.
6. **Report summary statistics and chromosome-level details.**

6. Impact of Parameter Settings and Handling of Ties

ExHap supports a number of user-configurable parameters. This algorithm was designed to identify allele identity across a large number of SNPs. The derivation window, specified by W, provides a "look ahead," favoring the selection of sequences that are identical beyond the recorded subsequence of length N. Commonly, we set W=30 and N=10.

The frequency of filtering, and thus elimination of non-congruent chromosomes, is user-configurable. For example, filtering can happen each iteration, or once every 10 iterations. Filtering each iteration removes failed chromosomes as quickly as possible, thereby eliminating them from subsequent computations of the consensus chromosome. Filtering less frequently increases processing speed. Additionally, the overlap of the filtering window is user-configurable.

In the event that there is a tie in the derivation of the evolving consensus chromosome, the first substring in alphabetical order is recorded (see Section 5, 3b above). In a study of 4 DQ-DR-B-A haplotype groups (3.8.1, 4.15.2, 4.44.2, and 7.57.1 consisting of 762, 337, 230 and 47 chromosomes respectively), across the 765 SNPs between *HLA-DRB1* and *HLA-B*, there were 1, 1, 4, and 5 ties, respectively. In all but one case, the recorded string was more specific than the tied string (e.g. TAACG instead of TxACG, where x represents an unknown/unphased allele). The more specific string is obviously preferred. In the event that tied strings are fully specified (all alleles one of A, C, G or T), selecting the first string provides a deterministic result. Since individual chromosomes are evaluated for identity with the consensus chromosome with some allowance for mismatch (e.g. 10 of 30 SNPs), we postulate that the selection of one substring over another probably does not unduly bias congruence calculations. Ties are reported in the system output (labeled with the phrase "TIE at final"), enabling users to perform their own analysis of the potential impact of these ties in their data sets.

The program can be executed in either a long-range or a rolling short-range mode. The long-range mode inspects chromosomes across hundreds or thousands of SNPs, such as the 1,818 SNPs between *HLA-DRB1* and *HLA-A*. This mode is useful when there is pre-existing knowledge of an extended haplotype, including approximate starting and ending points. The rolling short-range mode is combined with an overlapping sliding window to compute congruence on a smaller number of SNPs, e.g., 250. Then, analysis advances along the chromosome by an even smaller number of SNPs, e.g., 50. Both of these parameters are user-configurable. Congruence is computed again on the 250 SNPs until a large section of the chromosome has been analyzed, a small number of SNPs at a time. Then, for each window considered, we plot the percentage of congruent chromosomes for that window versus the base pair position of the window, as in Figures 5-8 of Baschal *et. al.* (Section 1.1). This mode is useful when there is not pre-existing knowledge of an extended haplotype, or when one wants to better analyze the boundaries of a particular extended haplotype.

7. Limitations

1. Input data must be comma delimited.
2. Alleles must be coded as 1, 2, 3, and 4.
3. The computation of congruence starts with the rightmost SNP in the specified range and moves to the leftmost. This is because the original data set focused on the MHC, on the short arm of chromosome 6, where congruence tends to be highest closest to the centromere and tends to deteriorate moving toward the telomere. In addition, the original set of SNPs were ordered from smallest base position to the largest.

8. Appendix: Configuration Properties in Alphabetical Order

Description	Property Name
The directory to which the allele output file should be written. The file names are <chromoTag>.<cohort>.summary.txt and <chromoTag>.<cohort>.alleles.txt<br><br>The directory name should end with a /	`alleleOutputDir`
The fully qualified path name for a comma delimited file containing SNP annotations. The structure of the file is <snp_name>,<text annotation>, as shown below:<br><br>`rs1059614,drb1`<br>`rs9266247,hla_b`<br>`rs1137078,hla_a`<br>`rs995185,UBD`<br><br>The order of the annotations does not need to correspond to the order of the SNPs in the chromosome file. However, only one line is allowed per SNP.	`annotationsFile`
The value in the field in the chromosome file specified by `caseFieldName` that represents "case." All other values will be counted as control.	`caseDesignator`
The label in the header row of the chromosome file that specifies the case-control field	`caseFieldName`

Description	Property Name
The starting SNP of the analysis region. ExHap assumes that the SNP data is arranged in order from lowest position (telomere) to highest position (centromere), moving from left to right. ExHap calculates the consensus from the centromere (rightmost SNP) to the telomere (leftmost SNP). The telomeric and centromeric terminology is based on the original application to the MHC, on the short arm of chromosome 6.	`centromericLocus`
The directory to which chromosome statistics are written.	`chromosomeStatsParentDir`
The phrase that will be part of the chromosome statistics file, and the chromosome statistics file name, e.g. "Conserved To A". Spaces in the phrase will be removed when the file name is created.	`chromoTag`
The path to and name of an allele summary file that contains a previously computed consensus sequence, used when `readConsensusFromFile=true`	`consensusFileName`
The maximum number of contiguous unphased/unknown chromosomes allowed.	`contiguousThold`
A true/false value indicating whether or not verbose output should be written to the output stream. This stream can be routed to a file.	`Debug`
The delimiter of the chromosome file and filter file (only comma(",") has been thoroughly tested)	`Delim`
The fully qualified path name (e.g. directory and file name) of the chromosome file	`filename`
A true/false value specifying whether the subset of chromosomes to be analyzed are specified by a list of fields embedded in the chromosome file	`filterByFields`
A comma delimited list of fields in the chromosome file to be used to define the filter (if `filterByFields=true`), e.g. filterFields=hla_drb_4digit,hla_dqa,hla_dqb_4digit	`filterFields`
The fully qualified path name (e.g. directory and file name) of the cohort filter file, to be used to filter the chromosome file based on cohort. Cohort refers to a subset of chromosomes (e.g. a haplotype group) that can be selected for analysis from a larger set of chromosomes.	`filterFile`
A comma delimited list of values in the chromosome file to match (if `filterByFields=true`), e.g. filterValues=1501,102,602	`filterValues`
The row number of the chromosome file on which the SNP names appear. Row numbers start at 1.	`headerRow`

Description	Property Name
The directory to which the heatmap files should be written. Note that the naming convention for the files is <cohort>.heatmap.txt, e.g. 3.8.1.heatmap.txt	`heatmapOutputDir`
The number of SNPs to record in the consensus pattern, and to step forward by (N, as described in Section 5)	`inspectionStep`
The character used to separate the values of the `labelParams`, e.g. "_"	`labelDelim`
A comma separated list of parameters to include in the custom row label (used when `outputCustomRowLabel=true`), e.g. snp,position,defaultMajorAllele	`labelParams`
The number of SNPs to inspect to compute a common pattern (W, as described in Section 5)	`lengthToInspect`
The row number of the chromosome file on which the Major Allele information appears. Row numbers start at 1. If the most common allele for a particular SNP is unphased or unknown, ExHap uses the major allele as the consensus value.	`majorAlleleRow`
The number of SNPs to consider when determining whether or not a chromosome is congruent with the consensus string (M, as described in Section 5).	`matchLength`
The number of positions to move forward before repeating the filtering out of non-matching chromosomes.	`matchStep`
The maximum number of mismatches allowed within the specified number of SNPs (L, as described in Section 5).	`mismatchLimit`
A true/false value indicating whether or not allele files should be output.	`outputAlleles`
A true/false value indicating whether or not annotations should be included in the heatmap and allele files	`outputAnnotations`
A true/false value controlling the presence of a custom row label in the heatmap and allele files. This label can be created from any combination and any order of numUnphased, snp, position, and defaultMajorAllele, e.g.<br><br>outputCustomRowLabels=true<br>labelParams=snp,position,defaultMajorAllele<br>labelDelim=_	`outputCustomRowLabels`
The percentage of unphased/unknown chromosomes allowed across the entire region.	`pctUnphasedThold`
The row number of the chromosome file on which the chromosome position appears. This value will be output as part of the heatmap files.	`positionRow`

**Description**	**Property Name**
A true/false value controlling the use of a consensus sequence that has been previously derived. This feature supports computation of consensus on one cohort, and then using that consensus sequence to calculate congruence metrics for a different cohort.	`readConsensusFromFile`
ExHap scores the remaining non-failed conserved chromosomes to see if they are still conserved based on this parameter. For example, scoreFrequency=1 scores the chromosomes every time they are inspected; scoreFrequency=5 scores the chromosomes every 5th inspection.	`scoreFrequency`
A value that indicates how the SNPs for telomericLocus and centromericLocus are specified. Options are:<br>byName<br>stringPosition<br>byBasePosition<br><br>byName means that the values will be something like rs389419<br>byBasePosition means the value with be something like 29468162<br><br>For either byName or byBasePosition, the values must be found in the appropriate header row of the chromsome file. Additionally, the value for the centromericLocus must be to the right of the value for the telomericLocus.<br><br>stringPosition refers to the column number in the chromosome file. This is useful for rolling congruence calculations, where ExHap is executed in a loop with telomericLocus and centromericLocus passed as properties on the command line.	`snpIdentification`
The ending SNP of the analysis region. ExHap assumes that the SNP data is arranged in order from lowest position (telomere) to highest position (centromere), moving from left to right. ExHap calculates the consensus from the centromere (rightmost SNP) to the telomere (leftmost SNP). The telomeric and centromeric terminology is based on the original application to the MHC, on the short arm of chromosome 6.	`telomericLocus`

Description	Property Name
The character to be used for textual representation of unphased/unknown alleles in the output. Unknown or unphased alleles in the chromsome input file must be represented as ? or N.	`unphased`
