# Supplementary material for: Congruence as a measurement of extended haplotype structure across the genome
Source: J Transl Med. 2012 Feb 27;10:32. doi: 10.1186/1479-5876-10-32 (PMC3310717; doi:10.1186/1479-5876-10-32)
Supplement: Additional file 5 — Figure S1. Congruence across the genome. These figures illustrate rolling short range congruence across chromosomes 1 through 22 for both CEU and YRI cohorts of the International HapMap Project. The horizontal reference lines (solid = CEH; dashed = YRI) show the 99th percentile of congruence (51.1% for CEU, 27.5% for YRI) for the respective cohorts. Overall, congruence was calculated for 27,652 overlapping ranges of 250 SNPs. Centromeres are indicated by "cen" at the starting position. Other loci of interest are indicated by the name [e.g. LCT or UNK (unknown)] and a source in which they are discussed (e.g. Sabeti2007). [file 1479-5876-10-32-S5.PDF]

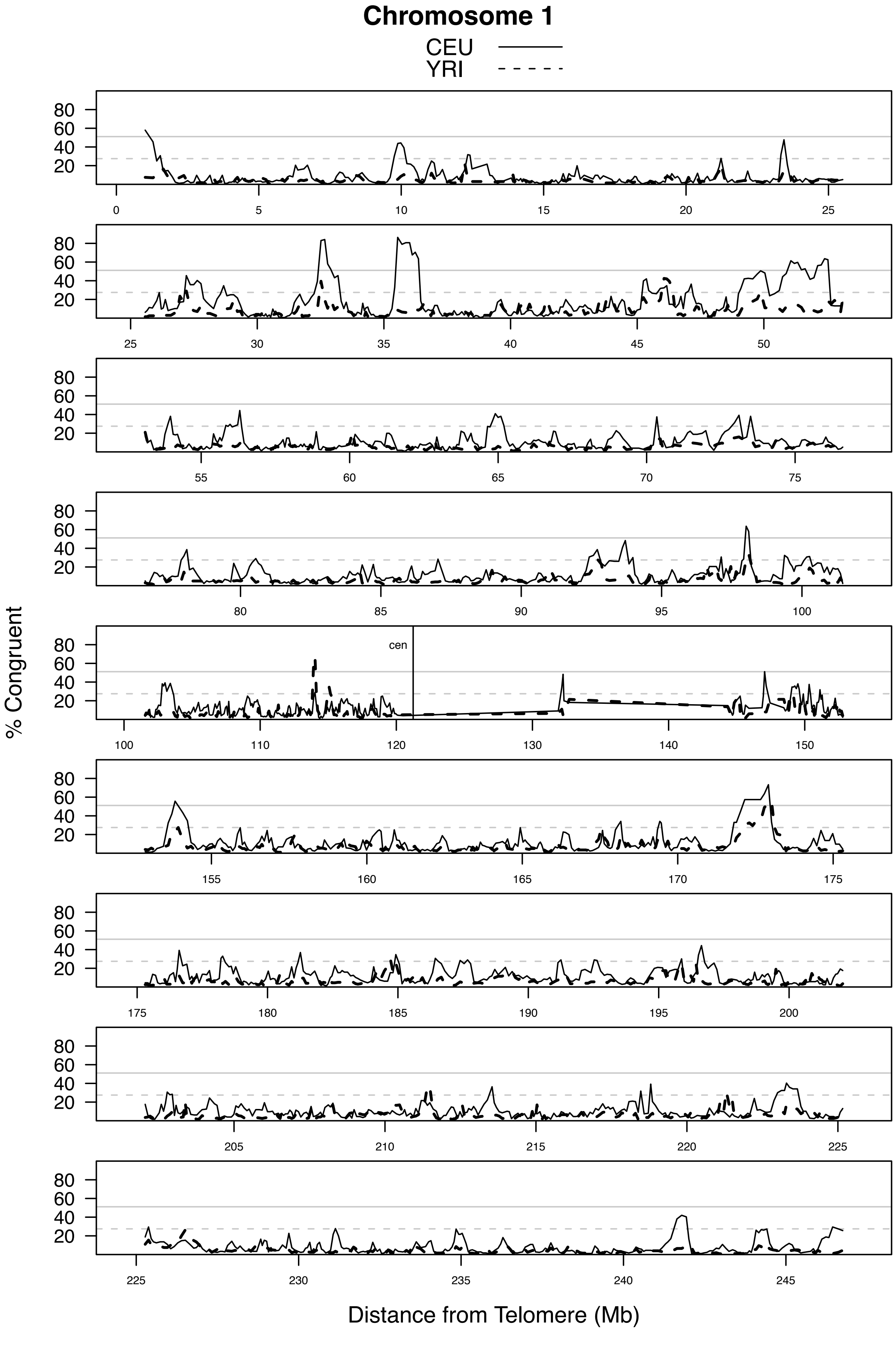
Chromosome 1
CEU
YRI
% Congruent
cen
Distance from Telomere (Mb)

Chromosome 2

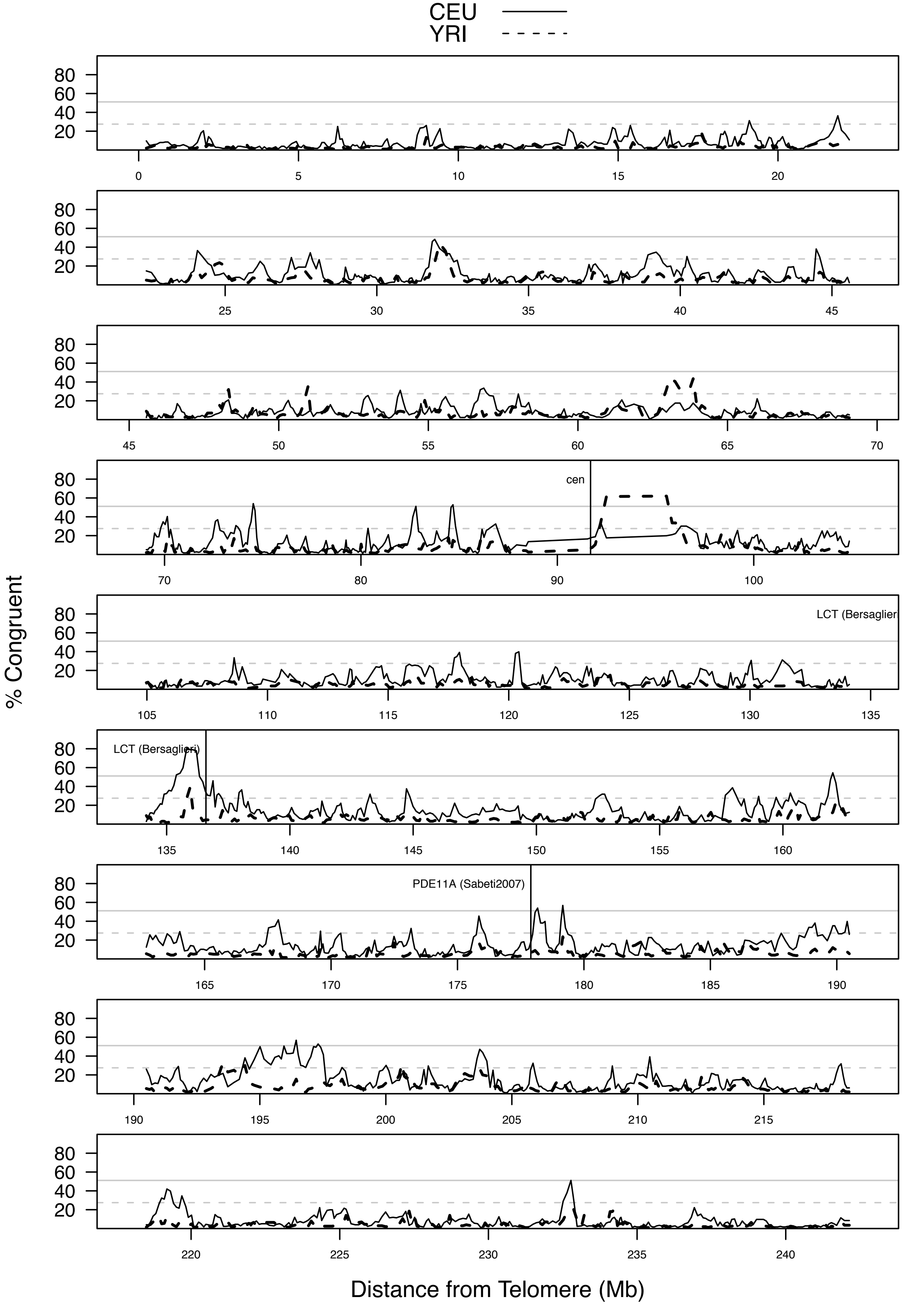

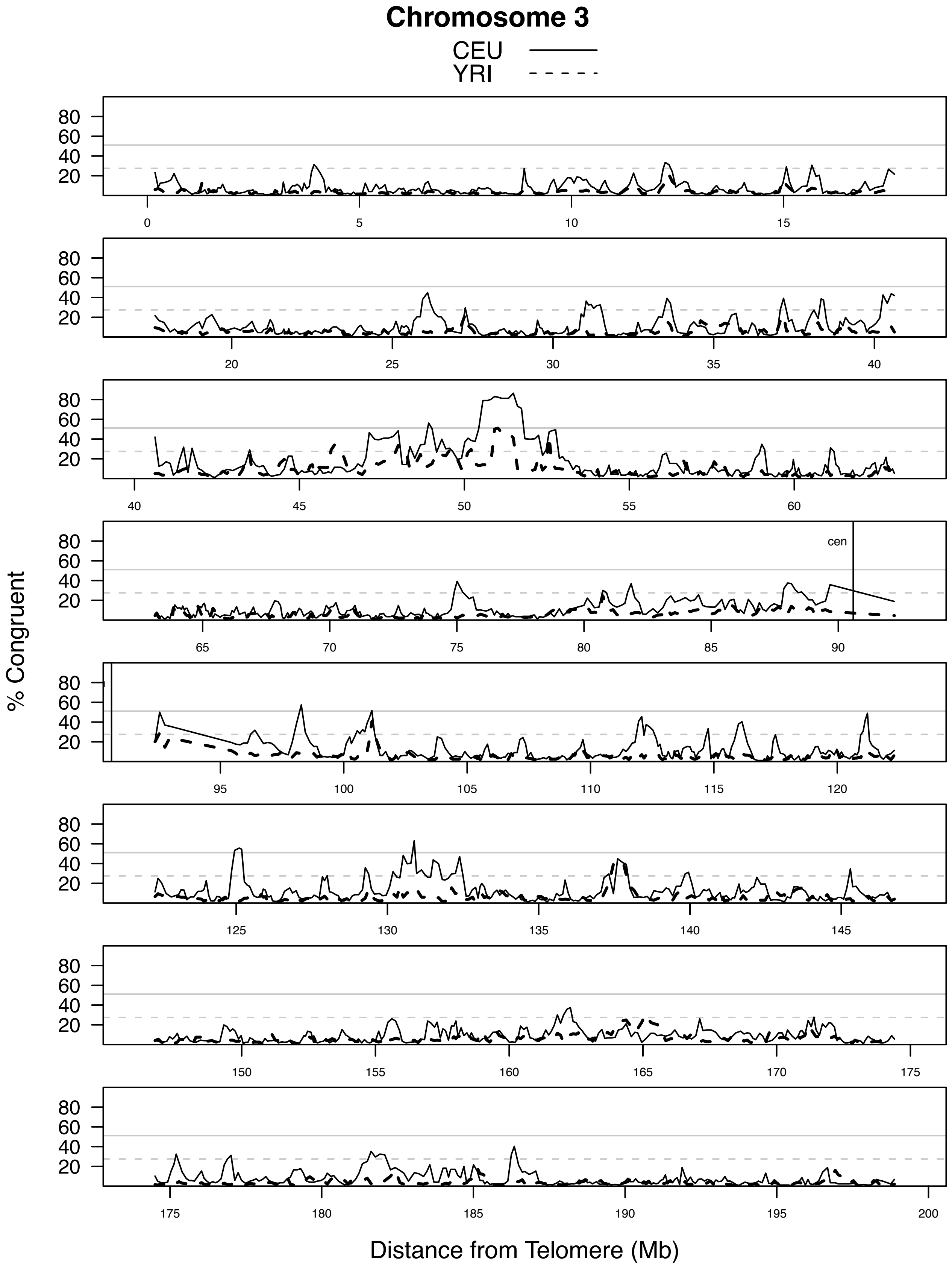

Chromosome 3
CEU
YRI
% Congruent
cen
Distance from Telomere (Mb)

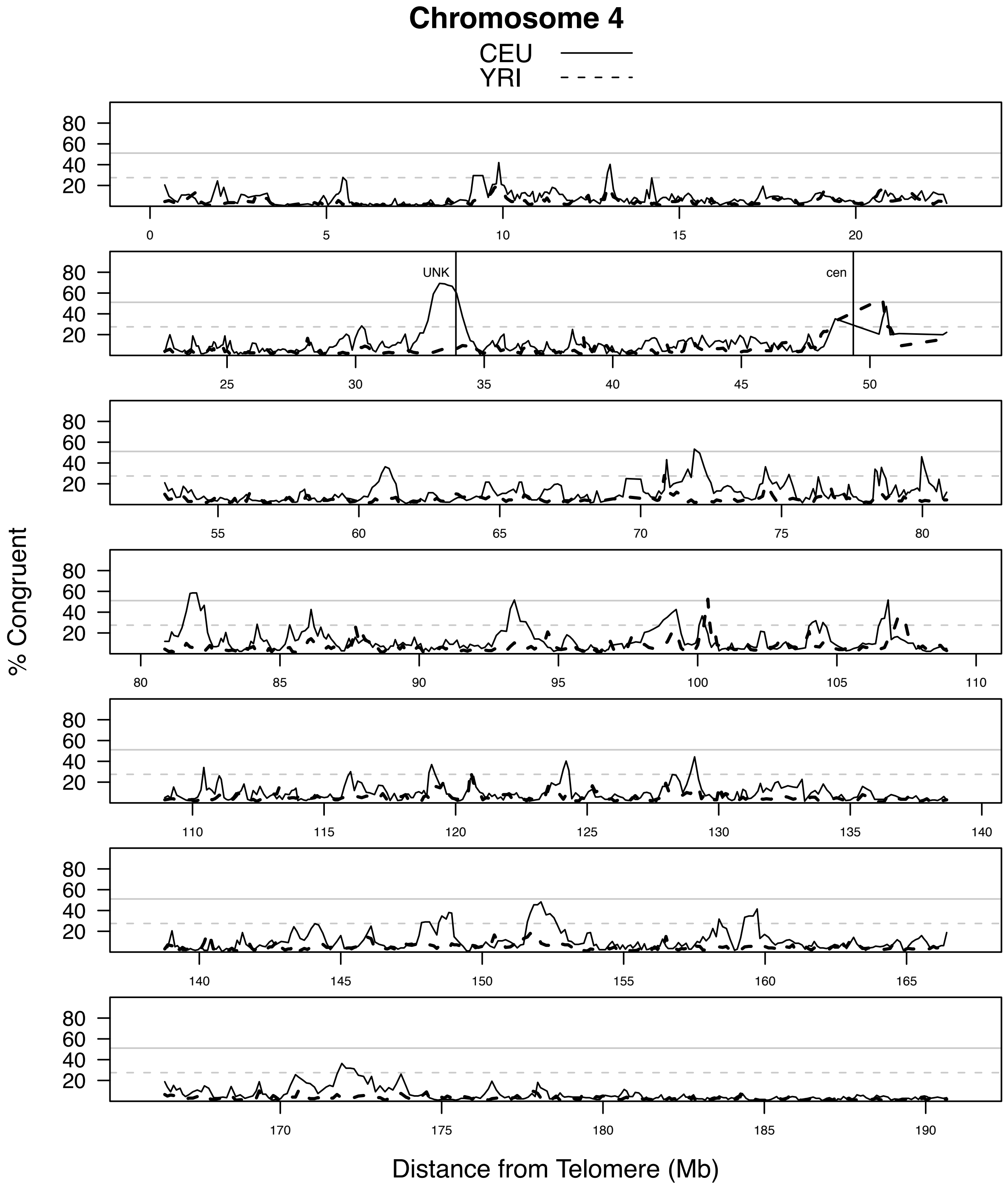
Chromosome 4
CEU
YRI
% Congruent
80
60
40
20
UNK
cen
Distance from Telomere (Mb)
0
5
10
15
20
25
30
35
40
45
50
55
60
65
70
75
80
85
90
95
100
105
110
115
120
125
130
135
140
145
150
155
160
165
170
175
180
185
190

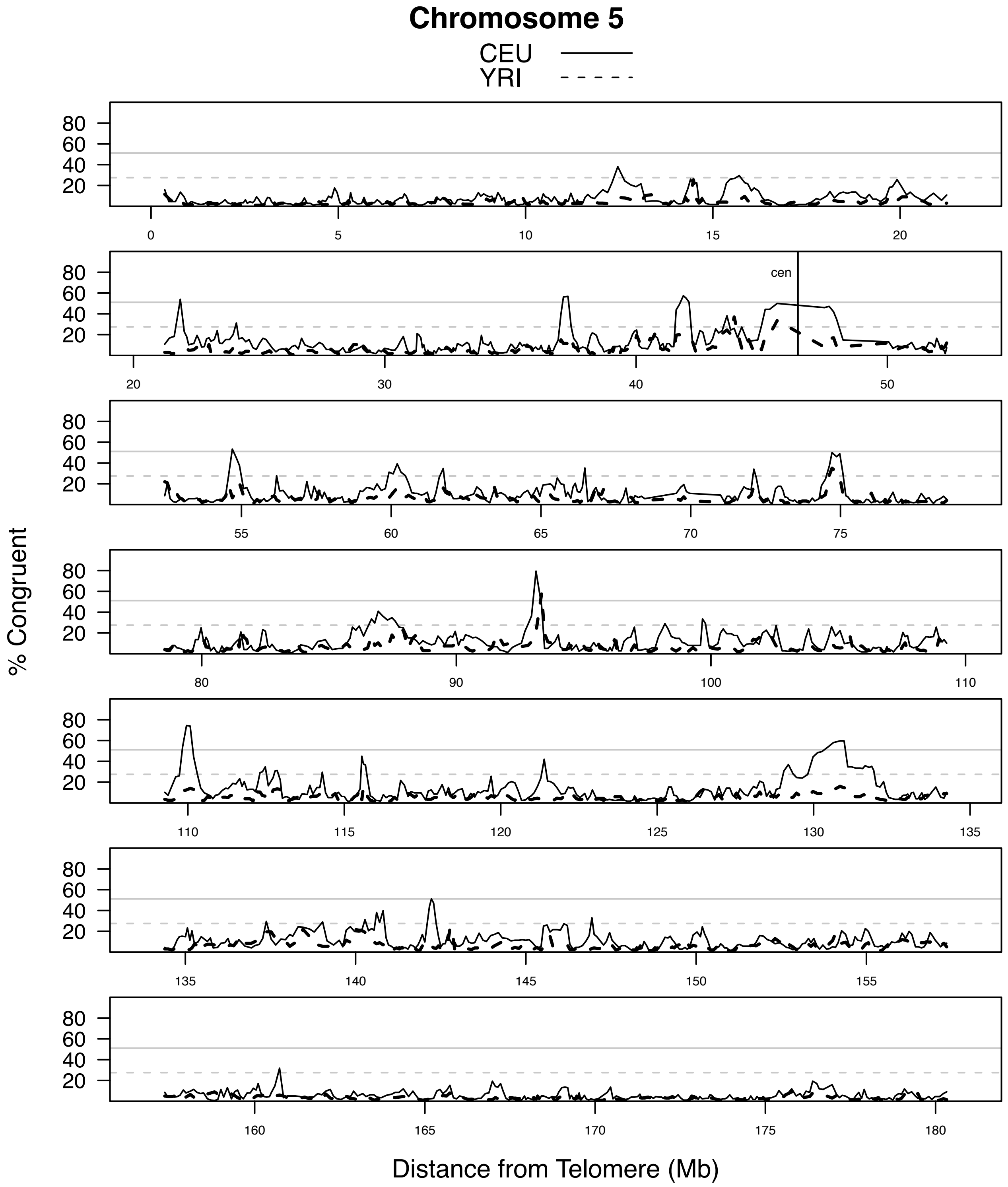
Chromosome 5
CEU
YRI
% Congruent
Distance from Telomere (Mb)
cen
80
60
40
20
0
5
10
15
20
30
40
50
55
60
65
70
75
80
90
100
110
115
120
125
130
135
140
145
150
155
160
165
170
175
180

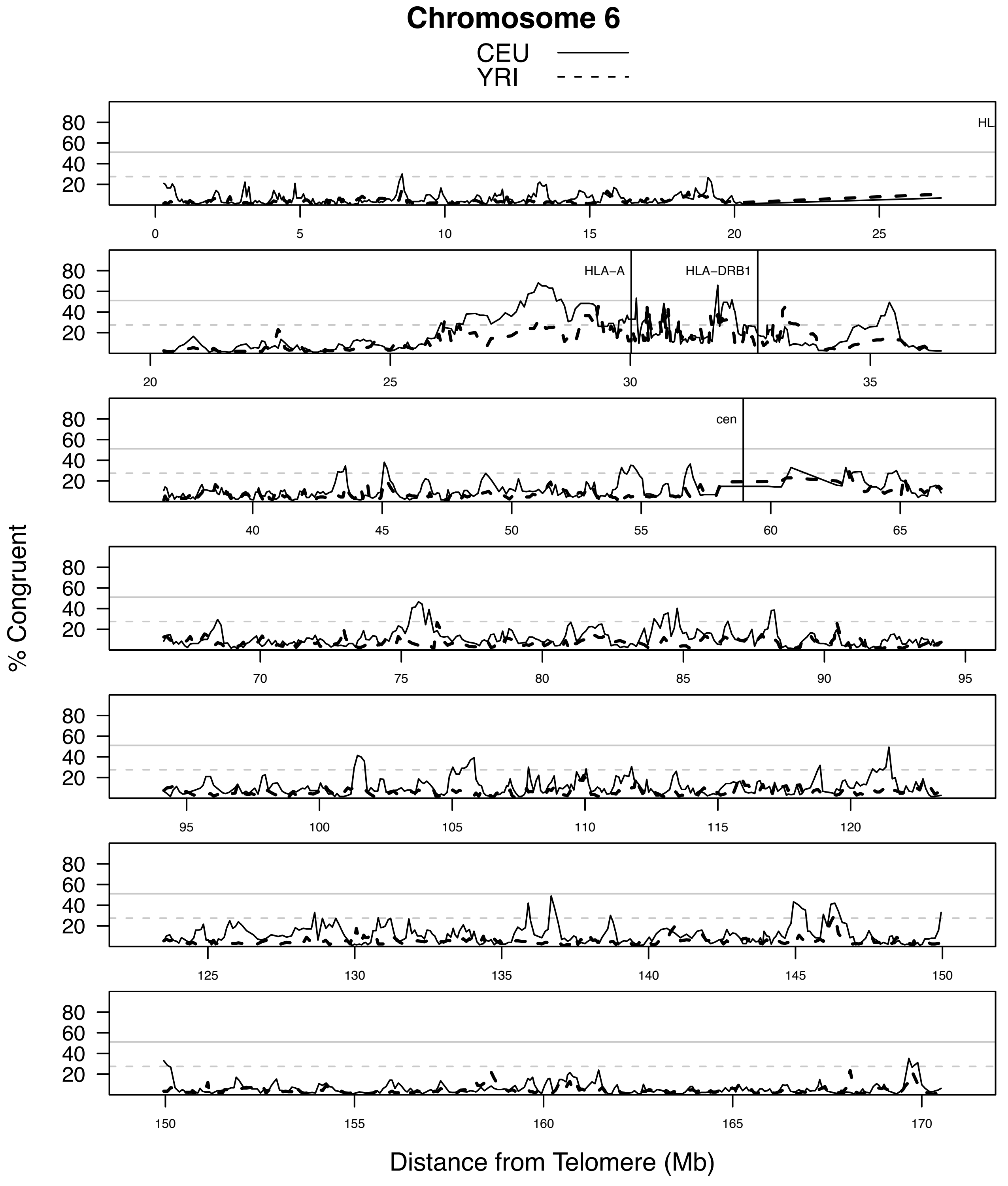
Chromosome 6
CEU
YRI
% Congruent
Distance from Telomere (Mb)
HL
HLA−A
HLA−DRB1
cen

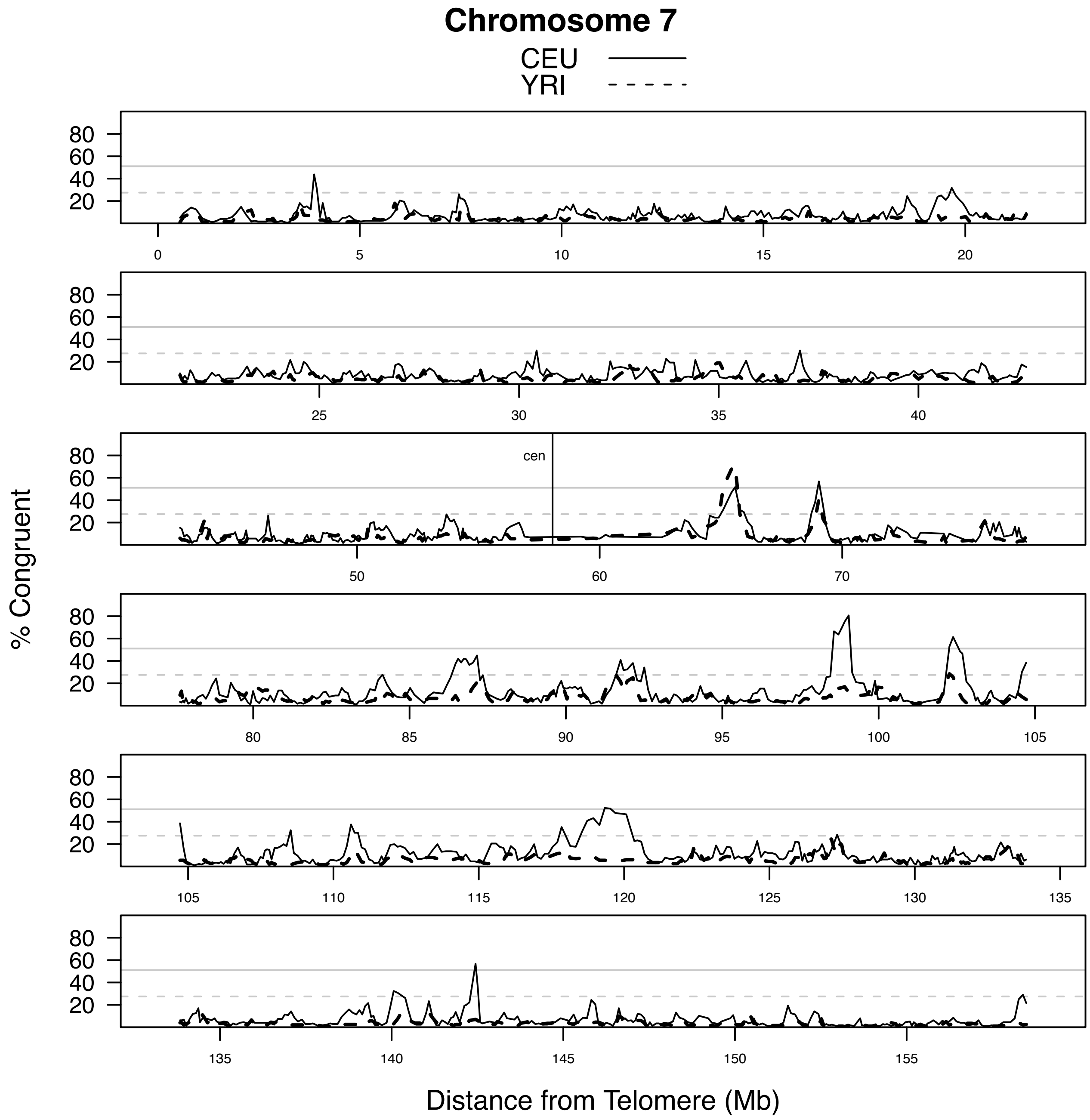
Chromosome 7
CEU
YRI
% Congruent
80
60
40
20
0
5
10
15
20
25
30
35
40
cen
50
60
70
80
85
90
95
100
105
110
115
120
125
130
135
140
145
150
155
Distance from Telomere (Mb)

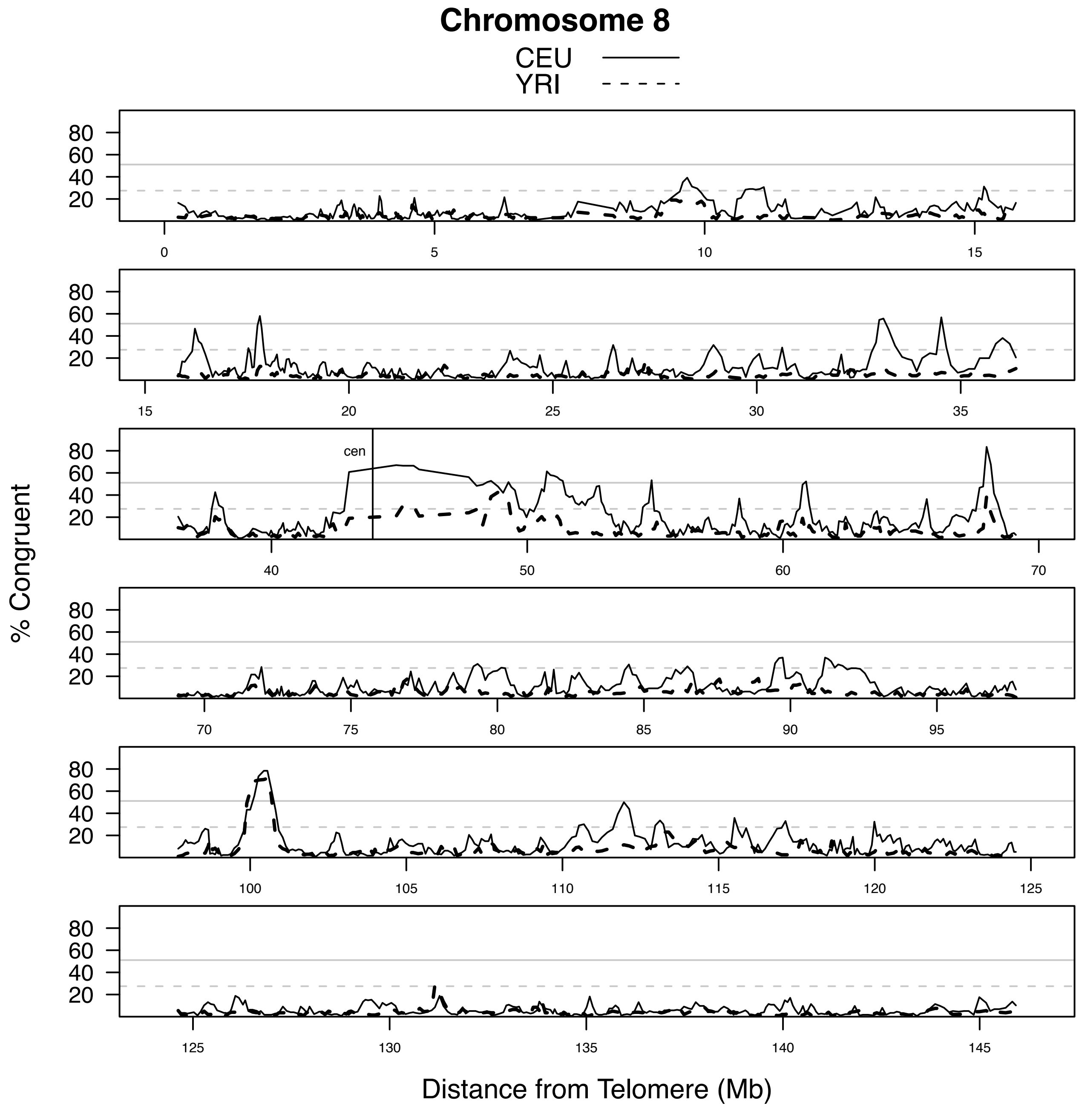

Chromosome 8
CEU
YRI
% Congruent
cen
Distance from Telomere (Mb)

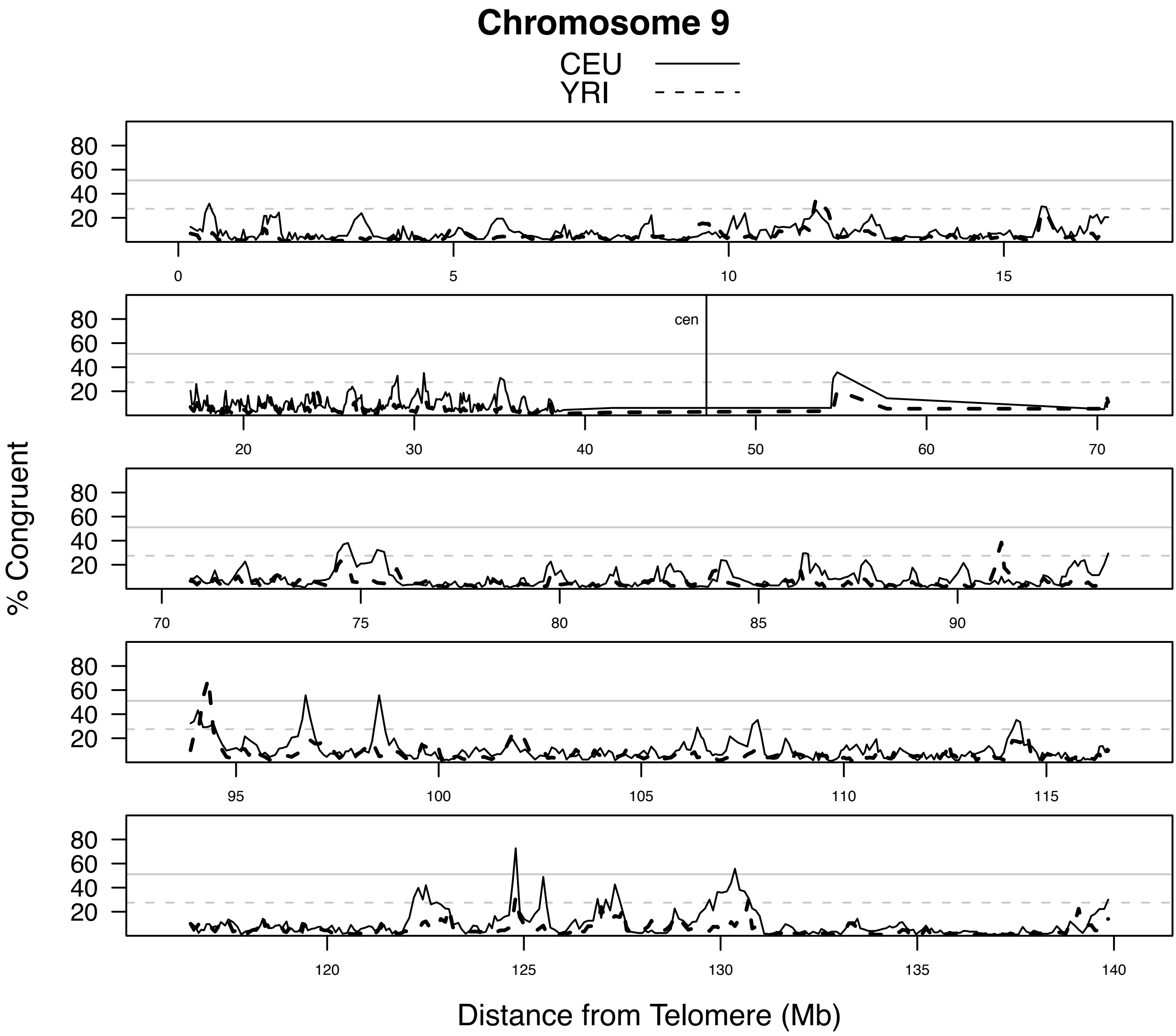
Chromosome 9
CEU
YRI
% Congruent
80
60
40
20
0
5
10
15
cen
20
30
40
50
60
70
70
75
80
85
90
95
100
105
110
115
120
125
130
135
140
Distance from Telomere (Mb)

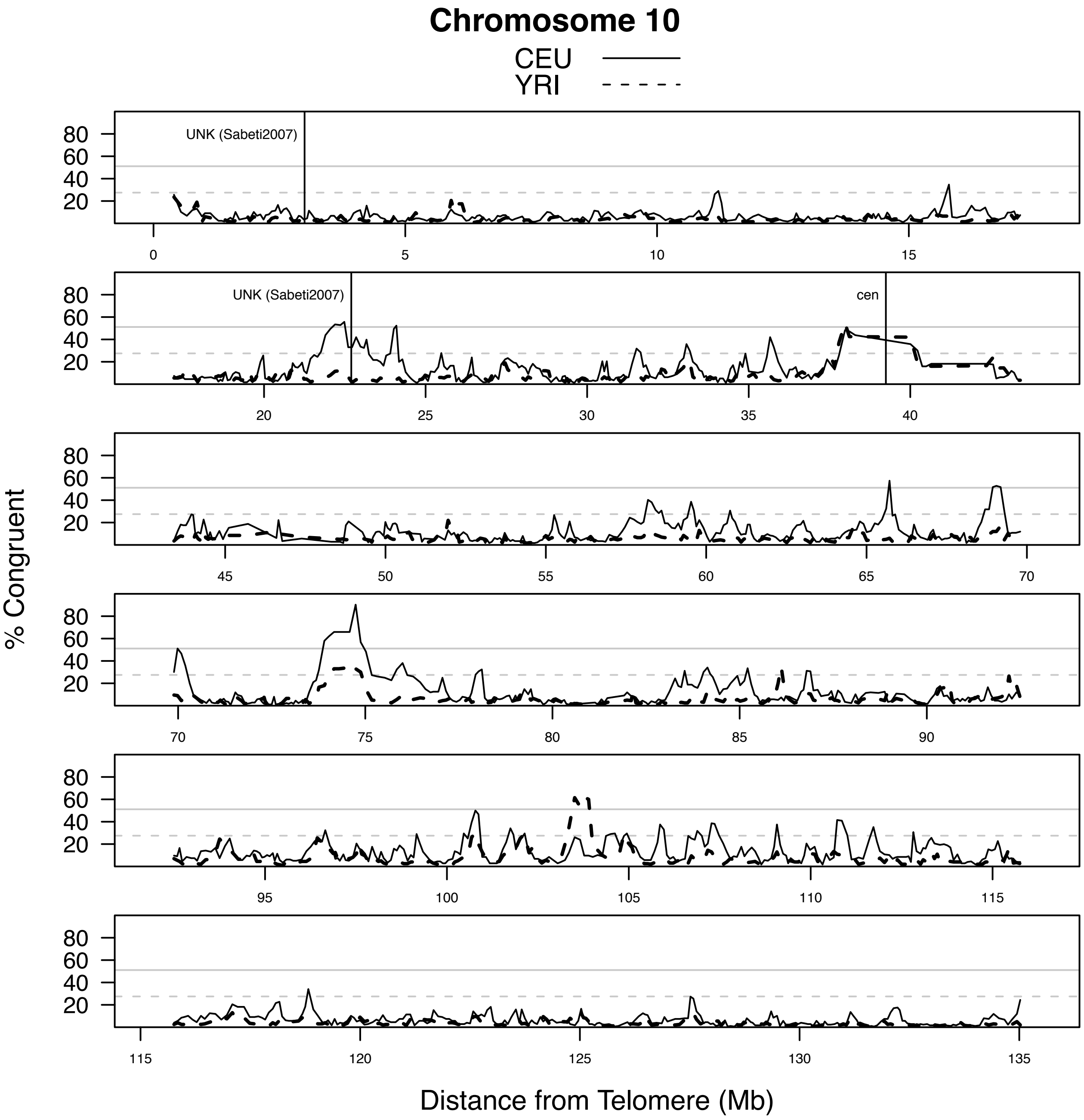
Chromosome 10
CEU
YRI
UNK (Sabeti2007)
UNK (Sabeti2007)
cen
% Congruent
Distance from Telomere (Mb)

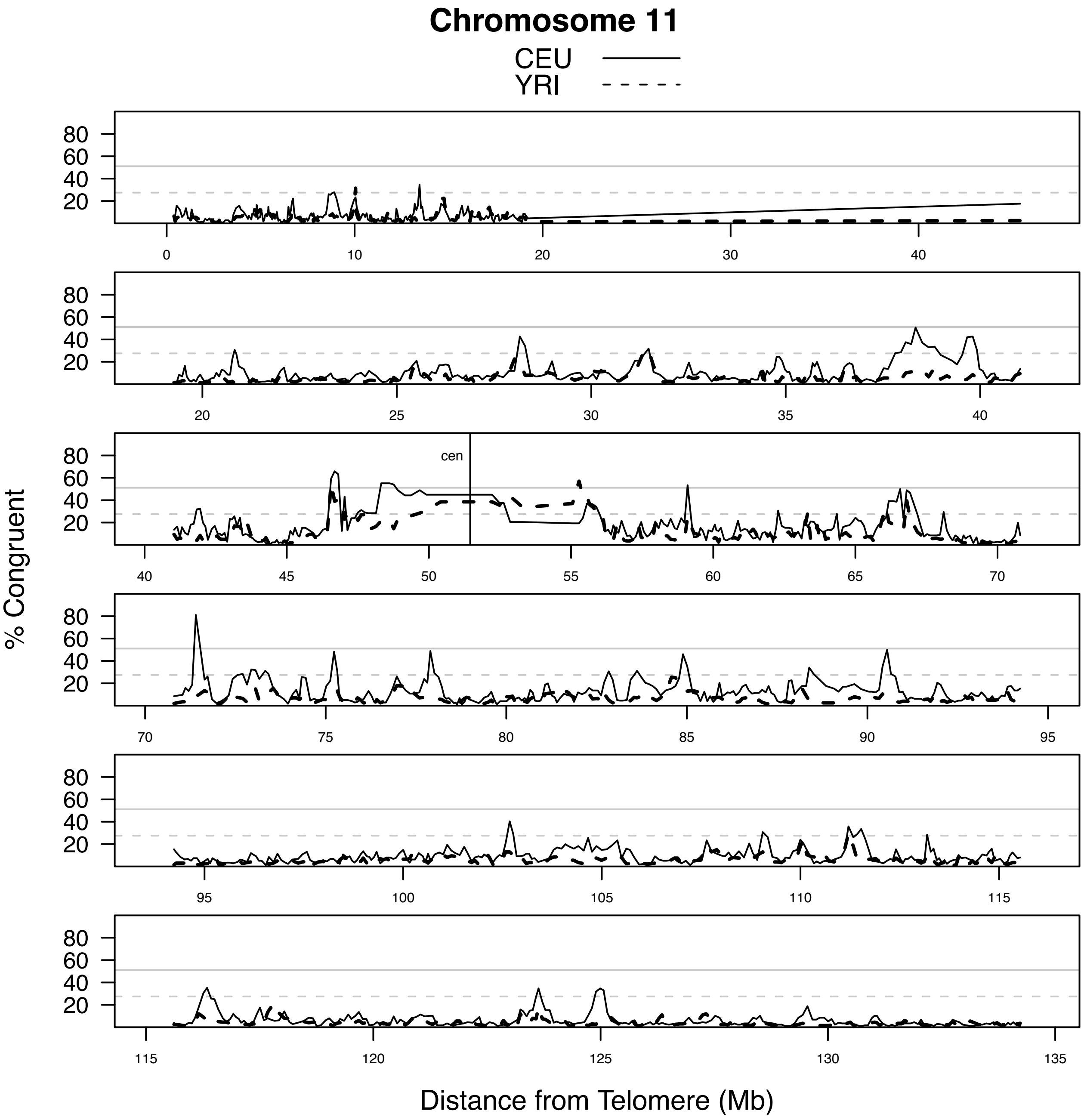

Chromosome 11
CEU
YRI
% Congruent
Distance from Telomere (Mb)
cen
80
60
40
20
0
10
20
30
40
25
35
45
50
55
60
65
70
75
80
85
90
95
100
105
110
115
120
125
130
135

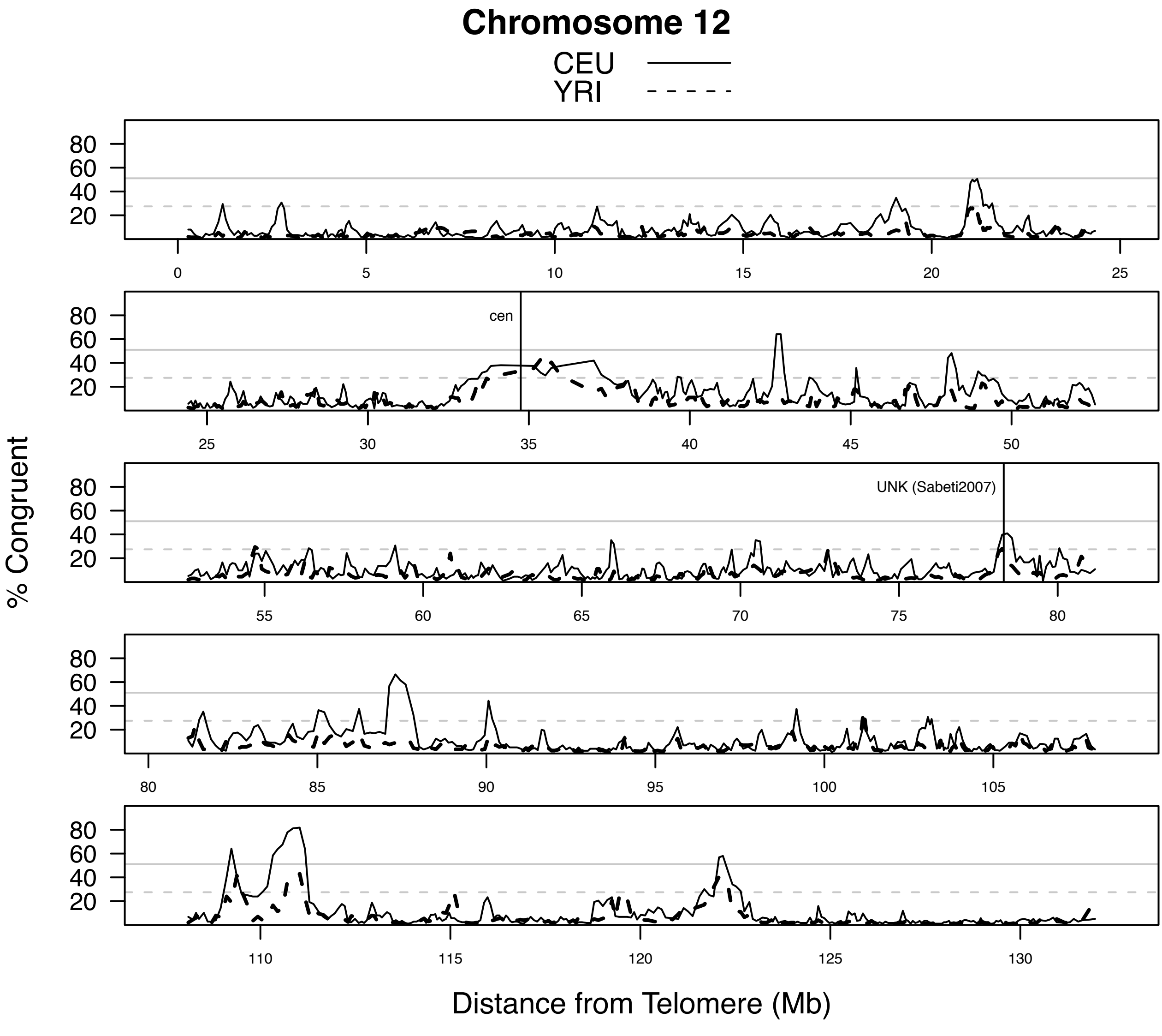
Chromosome 12
CEU
YRI
cen
UNK (Sabeti2007)
% Congruent
Distance from Telomere (Mb)

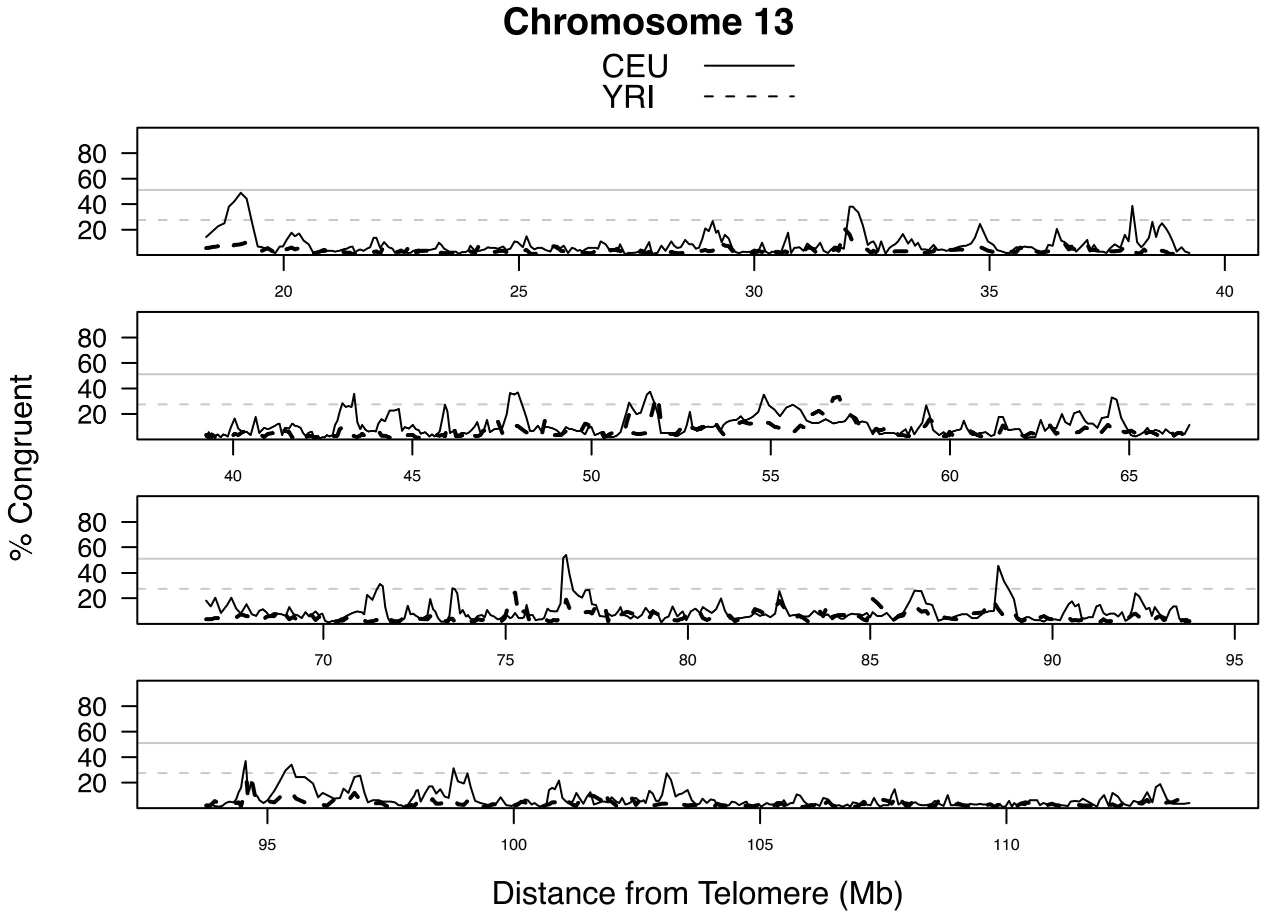

Chromosome 13
CEU
YRI
% Congruent
80
60
40
20
20
25
30
35
40
40
45
50
55
60
65
70
75
80
85
90
95
95
100
105
110
Distance from Telomere (Mb)

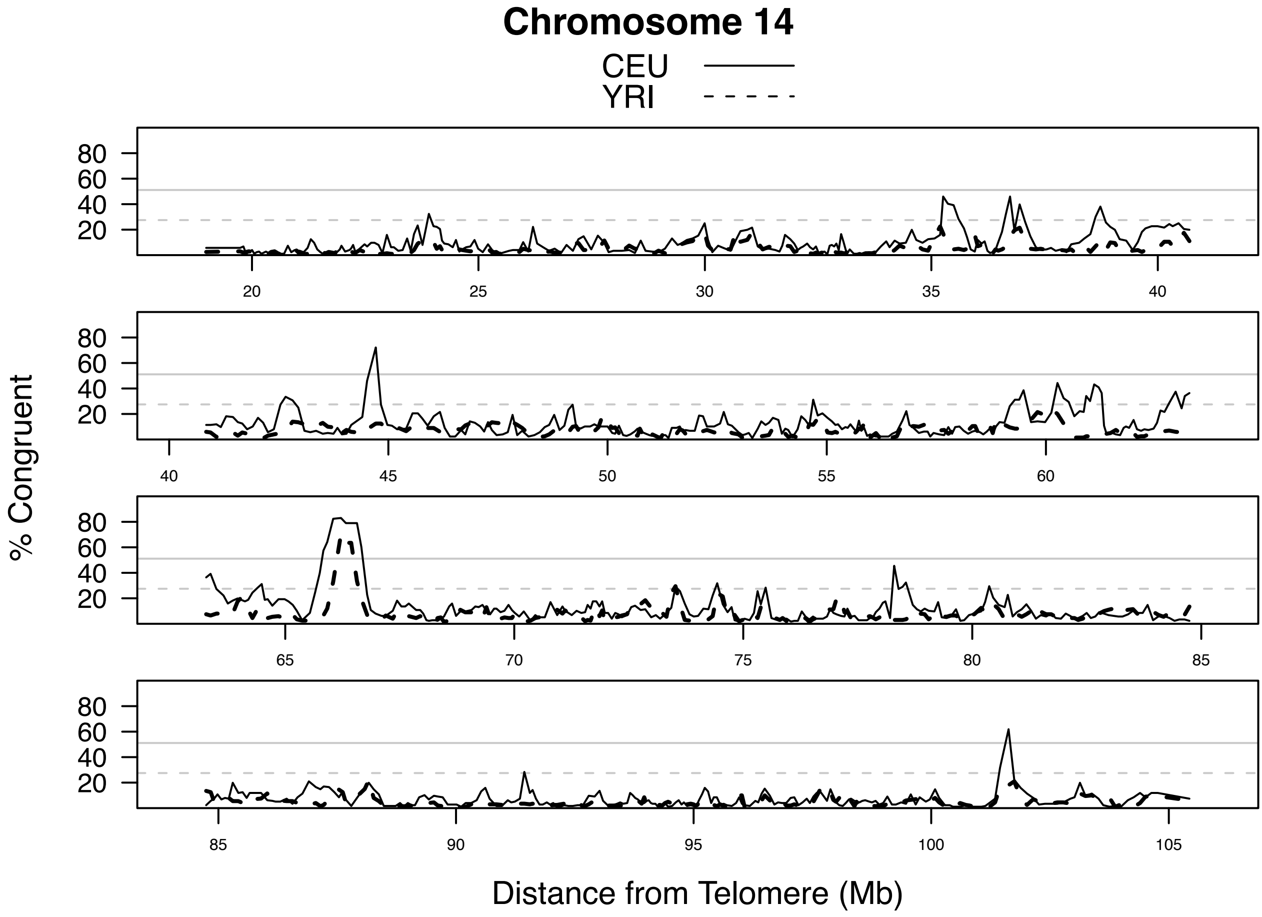

Chromosome 14
CEU
YRI
80
60
40
20
20
25
30
35
40
45
50
55
60
65
70
75
80
85
90
95
100
105
% Congruent
Distance from Telomere (Mb)

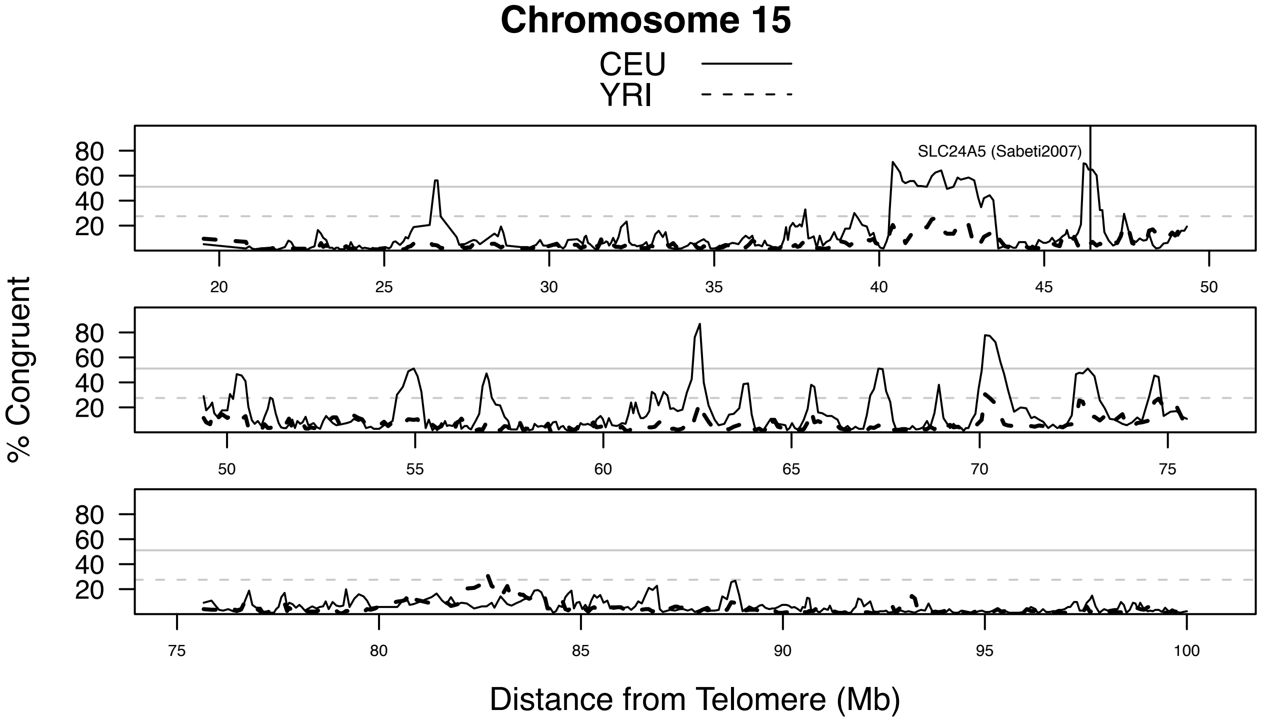
Chromosome 15
CEU
YRI
SLC24A5 (Sabeti2007)
% Congruent
80
60
40
20
20
25
30
35
40
45
50
55
60
65
70
75
80
85
90
95
100
Distance from Telomere (Mb)

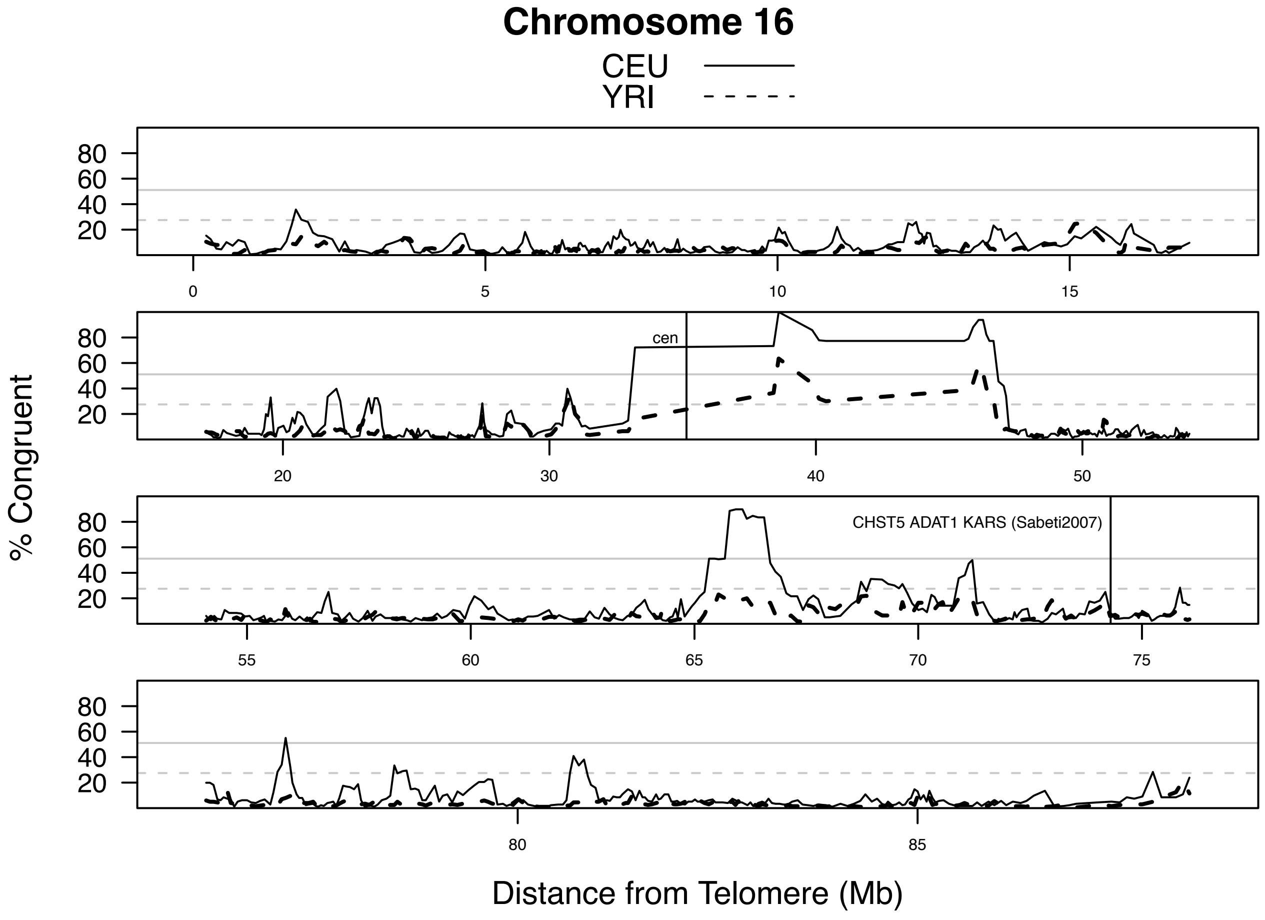

Chromosome 16
CEU
YRI
% Congruent
80
60
40
20
0
5
10
15
cen
20
30
40
50
CHST5 ADAT1 KARS (Sabeti2007)
55
60
65
70
75
80
85
Distance from Telomere (Mb)

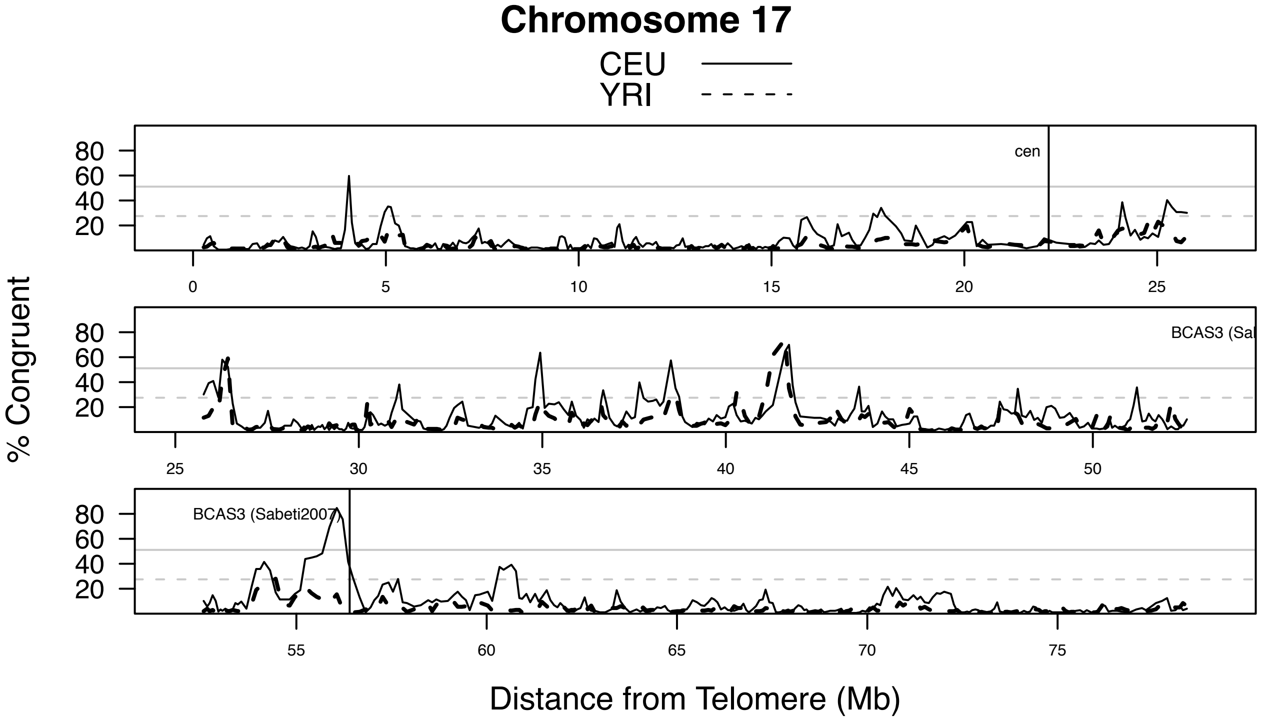
Chromosome 17
CEU
YRI
% Congruent
80
60
40
20
cen
0
5
10
15
20
25
BCAS3 (Sal
30
35
40
45
50
BCAS3 (Sabeti2007)
55
60
65
70
75
Distance from Telomere (Mb)

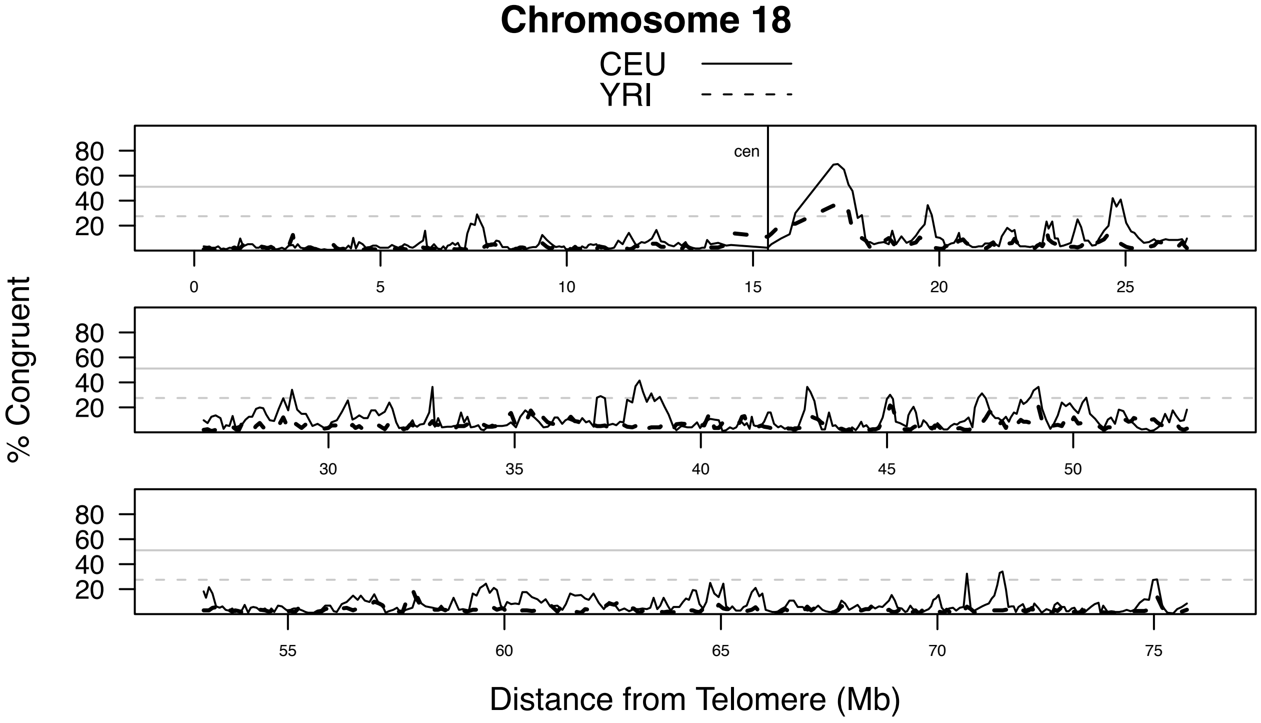
Chromosome 18
CEU
YRI
cen
80
60
40
20
0
5
10
15
20
25
30
35
40
45
50
55
60
65
70
75
% Congruent
Distance from Telomere (Mb)

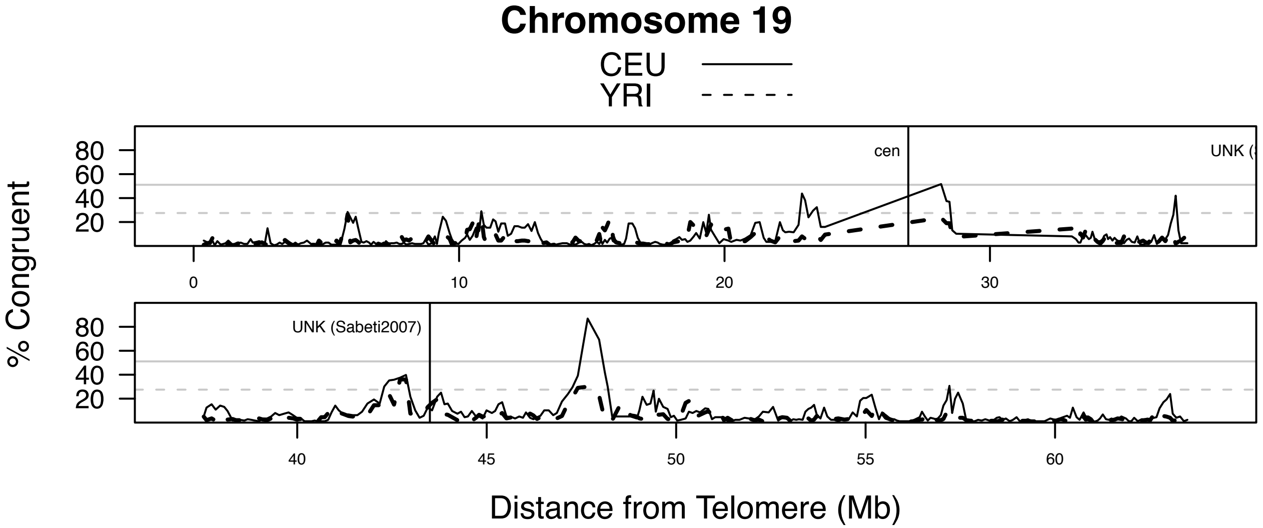

Chromosome 19
CEU
YRI
% Congruent
80
60
40
20
cen
UNK (S
0
10
20
30
UNK (Sabeti2007)
40
45
50
55
60
Distance from Telomere (Mb)

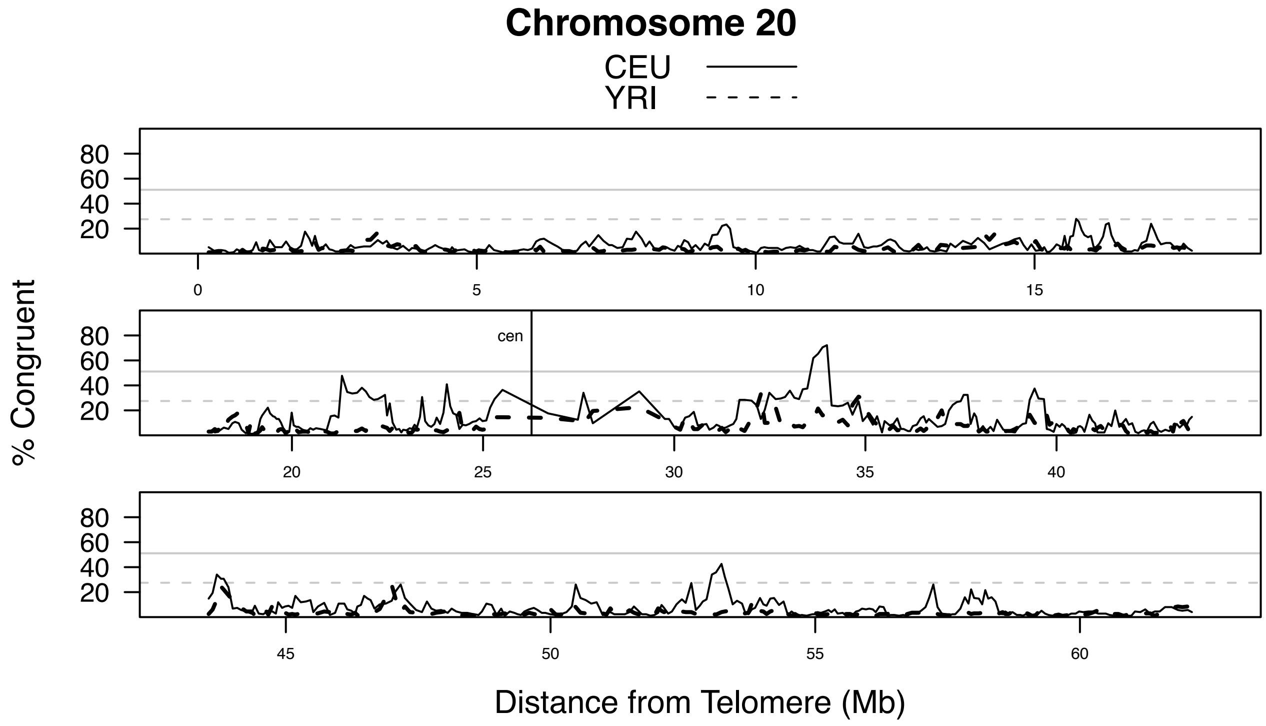

Chromosome 20
CEU
YRI
80
60
40
20
0
5
10
15
cen
20
25
30
35
40
45
50
55
60
% Congruent
Distance from Telomere (Mb)

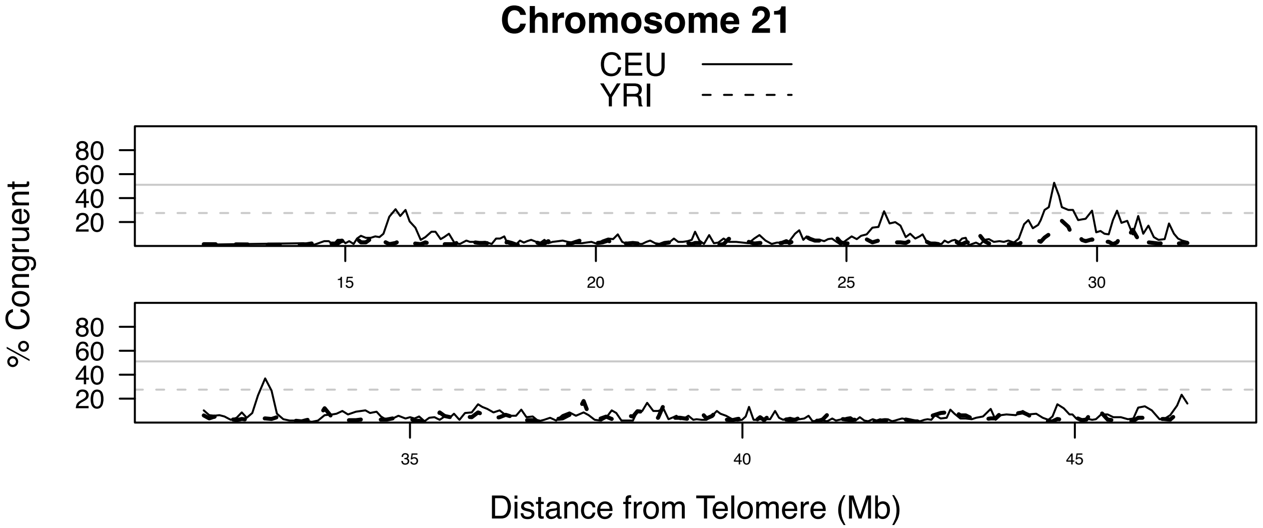

Chromosome 21
CEU
YRI
% Congruent
80
60
40
20
15
20
25
30
80
60
40
20
35
40
45
Distance from Telomere (Mb)

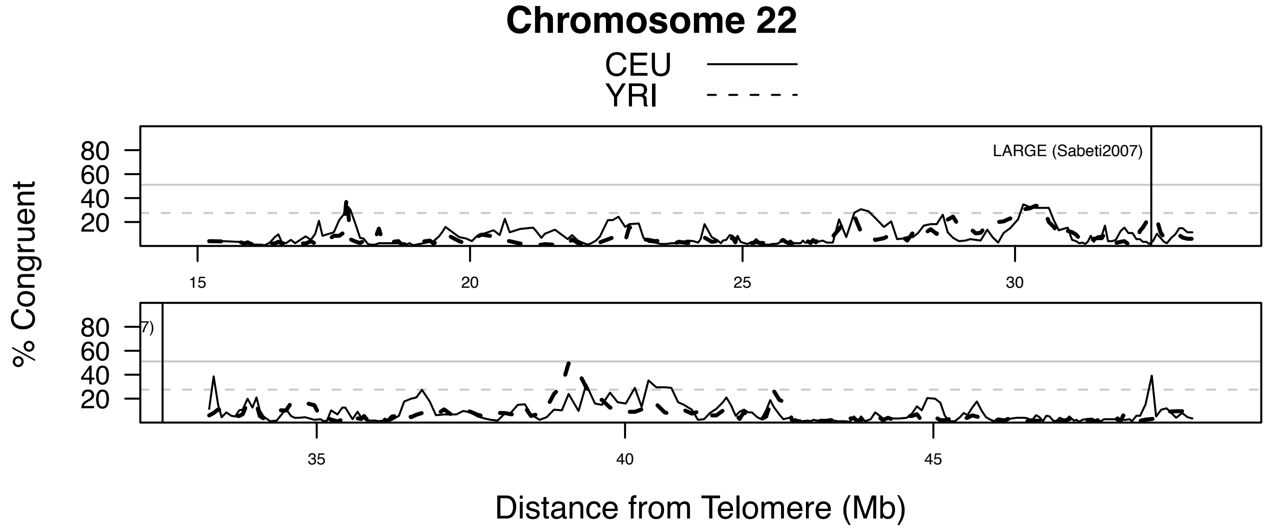

Chromosome 22
CEU
YRI
% Congruent
80
60
40
20
LARGE (Sabeti2007)
15
20
25
30
7)
35
40
45
Distance from Telomere (Mb)
