# Supplementary figures and images for: Congruence as a measurement of extended haplotype structure across the genome
Source: J Transl Med. 2012 Feb 27;10:32. doi: 10.1186/1479-5876-10-32 (PMC3310717; doi:10.1186/1479-5876-10-32)

## Chromosome 10

### Congruence vs. Recombination Rate

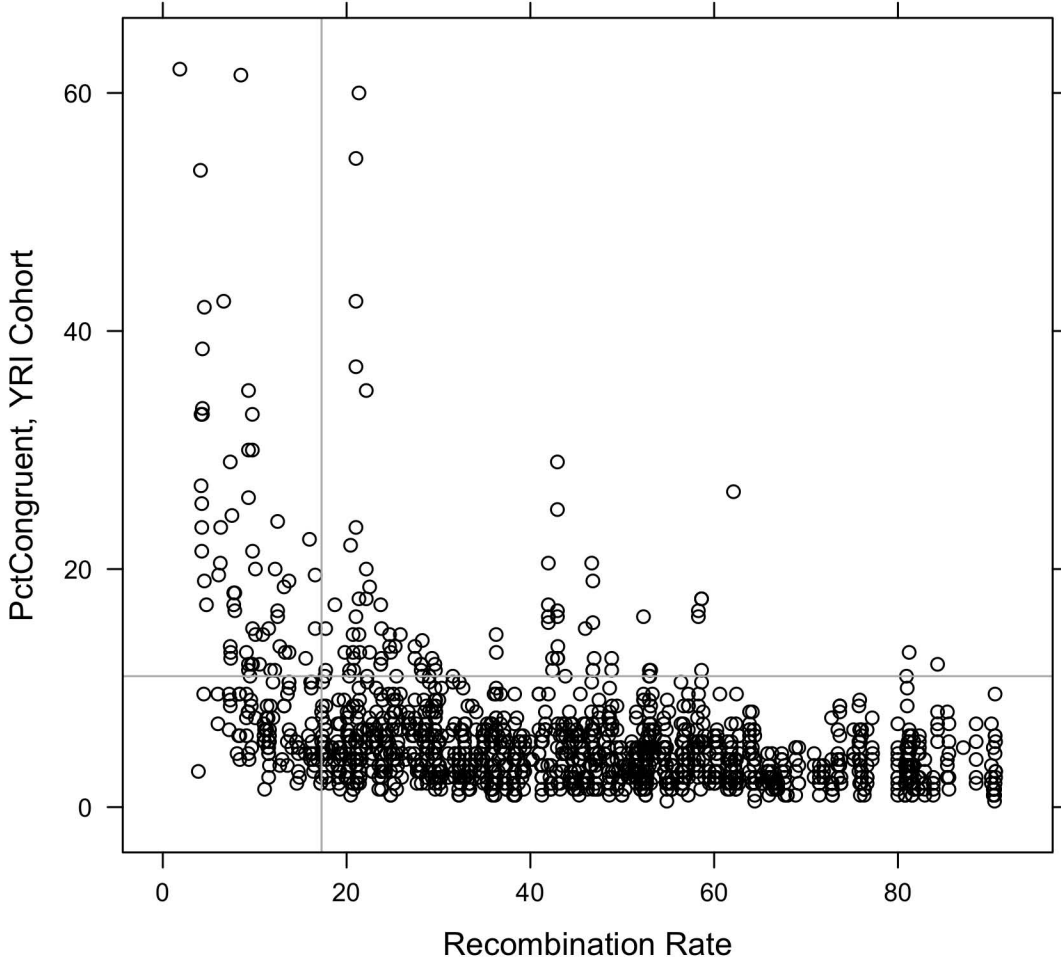

Supplement: Additional file 8 — Figure S2. For chromosome 10, the maximum recombination rate was identified for each 250 SNP range for which congruence was calculated. These recombination rates are plotted against the corresponding congruence rates for the YRI cohort. Reference lines indicate the 90th percentile of the congruence rates (11%) and the 10th percentile of the recombination rates (17%). The points in the upper right hand quadrant represent those regions for which both congruence and recombination are relatively high and potentially warrant further investigation. [file 1479-5876-10-32-S8.PDF]
